# Dynamic relationships between spontaneous and evoked electrophysiological activity

**DOI:** 10.1038/s42003-021-02240-9

**Published:** 2021-06-15

**Authors:** Soren Wainio-Theberge, Annemarie Wolff, Georg Northoff

**Affiliations:** 1 Mind, Brain Imaging and Neuroethics, Institute of Mental Health Research, University of Ottawa, Ottawa, ON Canada; 2 Integrated Program in Neuroscience, McGill University, Montréal, QC Canada; 3 Mental Health Centre, Zhejiang University School of Medicine, Hangzhou, Zhejiang China

**Keywords:** Neuroscience, Perception, Consciousness

## Abstract

Spontaneous neural activity fluctuations have been shown to influence trial-by-trial variation in perceptual, cognitive, and behavioral outcomes. However, the complex electrophysiological mechanisms by which these fluctuations shape stimulus-evoked neural activity remain largely to be explored. Employing a large-scale magnetoencephalographic dataset and an electroencephalographic replication dataset, we investigate the relationship between spontaneous and evoked neural activity across a range of electrophysiological variables. We observe that for high-frequency activity, high pre-stimulus amplitudes lead to greater evoked desynchronization, while for low frequencies, high pre-stimulus amplitudes induce larger degrees of event-related synchronization. We further decompose electrophysiological power into oscillatory and scale-free components, demonstrating different patterns of spontaneous-evoked correlation for each component. Finally, we find correlations between spontaneous and evoked time-domain electrophysiological signals. Overall, we demonstrate that the dynamics of multiple electrophysiological variables exhibit distinct relationships between their spontaneous and evoked activity, a result which carries implications for experimental design and analysis in non-invasive electrophysiology.

## Abstract (web summary)

Wainio-Theberge et al employed a large-scale magnetoencephalographic dataset and an electroencephalographic replication dataset to investigate the relationship between spontaneous and evoked neural activity across a range of electrophysiological variables. They demonstrated that the dynamics of multiple electrophysiological variables exhibit distinct relationships between their spontaneous and evoked activity, which carries implications for experimental design and analysis.

## Introduction

Even in the absence of specific experimental stimulation, neural activity displays spontaneous fluctuations with a characteristic temporal and spatial structure^1,2^. This spontaneous neural activity has been associated with various forms of internally-oriented cognition, such as mind wandering^3,4^, self-referential processing^5–7^, mental time travel^8–10^ and social cognition/theory of mind^11^. While spontaneous activity is typically studied in stimulus-free “resting-state” designs, it persists in cognitive tasks as trial-by-trial fluctuations in neural activity. Multiple recent studies have now demonstrated that spontaneous neural activity prior to stimulus onset can predict or influence the subject’s stimulus-related perception^12–14^, sense of self^15,16^, consciousness^17–21^, attention^22^, reaction time^23^, and working memory^24^. This pervasive influence of pre-stimulus activity on behavioral outcomes raises questions about the neural mechanism of this effect. If spontaneous pre-stimulus neural activity is to have behaviorally observable effects, then it must influence the brain’s processing of external stimuli, and hence also influence neural activity changes following stimulus onset. However, these dynamic relationships between spontaneous pre-stimulus and stimulus-evoked activity remain poorly understood.

Previous small-sample functional Magnetic Resonance Imaging (fMRI) studies have revealed a negative correlation between spontaneous and evoked Blood Oxygen Level Dependent signal (BOLD) amplitudes^25,26^. In EEG investigations, the power of cortical oscillations, in particular the alpha band, are thought to reflect modulations of cortical excitability, and as such have been investigated for their role in shaping stimulus processing^27–32^. Other work has investigated the influence of more general physiological variables such as desynchronization and arousal on poststimulus activity, operationalizing these in various ways^33–35^, or has conducted modeling work on the question^36,37^. While this work has revealed useful insights into the relationship between ongoing brain states and stimulus processing, the specific electrophysiological variables which exhibit relationships between their ongoing and evoked dynamics are not known, nor are the form of these relationships (e.g., positive versus negative correlation) clear.

Electroencephalography (EEG) and magnetoencephalography (MEG) studies on the relationship of spontaneous and evoked activity have typically focussed on different facets of neural physiology on either side of stimulus onset, selecting particular operational measures of pre-stimulus activity based on hypotheses about more general physiological variables, such as desynchronization. However, it remains unknown whether there are systematic relationships between spontaneous and evoked activity within the same electrophysiological features, rather than both components being operationalized by distinct measures. Are there, for instance, dynamic mechanisms by which spontaneous pre-stimulus variation in gamma power affects stimulus-evoked gamma responses, or, alternatively, are evoked responses influenced primarily by more general brain states of excitability and synchronization? While such a spontaneous-evoked relationship has been shown in the dynamics of the BOLD signal in fMRI^25,26^, it has never been investigated in MEG/EEG. This is of considerable importance as the physiological interpretation of the MEG/EEG signal is more feasible and includes a greater diversity of neurophysiological processes.

EEG and MEG provide a window into an abundance of neurophysiological phenomena^38^, and analyses in the time and frequency domains reflect different underlying neurophysiological processes, though these relationships are not necessarily specific or fully understood. The time-domain electrophysiological signal is known to reflect synchronous postsynaptic potentials of many neurons^39^, while frequency domain analyses allow one to record cortical oscillations such as alpha (8–13 Hz), theta (4–8 Hz), and beta (13–25 Hz);^38^ these are thought to reflect cortical feedback loops or neurotransmitter-related processes^40,41^. Frequency-domain analyses also reveal arrhythmic “scale-free” activity, which has been associated with excitation-inhibition balance^42,43^ and complex network models of self-organized criticality^44,45^. Though many of these neurophysiological parameters have been investigated in terms of their pre-stimulus or stimulus-evoked activity, it remains unknown which of them, if any, shows a correlation between spontaneous pre-stimulus and evoked activity.

In the present study, we investigate the relationship between spontaneous and stimulus-evoked neural activity for a diverse set of electrophysiological variables. For this purpose, we employ a large-scale MEG data set with a simple sensory paradigm^46,47^, as well as a replication EEG dataset with a more complex cognitive task;^48^ this allowed us to probe for task-specificity vs. -unspecificity of the pre-stimulus and stimulus-evoked relationship. In the frequency domain, we observed widespread correlation between spontaneous and evoked spectral power in multiple frequency bands, a finding which was consistent across modalities and tasks; however, the type and magnitude of this correlation varied between frequency bands. Disentangling the contributions of different physiological sources of spectral power, we found that the correlations of spontaneous and evoked activity found in the mixed power were largely recapitulated when examining purely oscillatory power. Two parameters of scale-free activity (the scaling exponent and broadband offset) also showed positive correlations between their spontaneous and evoked dynamics. Correlations between spontaneous and evoked activity were also observed in the time domain, where they appeared to be task- or modality-specific. Our study sheds new light on the different relationships between spontaneous and evoked neural activity of numerous common electrophysiological variables. In turn, this may in future help explain the neurophysiological mechanism by which trial-by-trial fluctuations in spontaneous activity affect cognitive and perceptual outcomes.

## Results

**Fig. 1:**
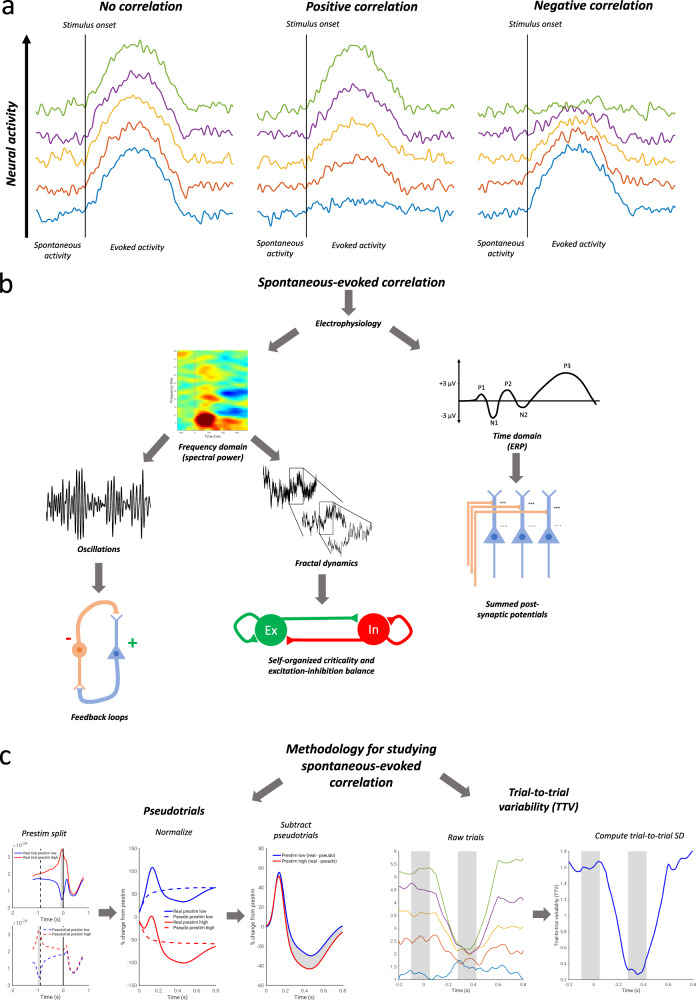
Schematic of the main aims and methods of the study. **a** Schematic of different interaction schemes between spontaneous and evoked activity. With no spontaneous-evoked correlation, evoked amplitudes are identical regardless of the level of prestimulus spontaneous activity. With positive correlation, higher prestimulus spontaneous activity leads to greater evoked amplitudes. With negative correlation, higher prestimulus spontaneous activity leads to lower evoked amplitudes. **b** Aims of the study. The study aims to assess which electrophysiological processes exhibit relationships between spontaneous prestimulus and evoked poststimulus activity. The study considers electrophysiological dynamics in the time and frequency domains, further classifying frequency domain evoked power as reflecting oscillations or scale-free (fractal) dynamics. Each of these electrophysiological parameters is associated with different physiological processes. **c** Methodology for assessing spontaneous-evoked correlation. In the method of pseudotrials, trials and pseudotrials are split into prestimulus high and low conditions. They are then normalized relative to the mean prestimulus period, and the pseudotrial time courses are subtracted: any difference is indicative of a relationship between spontaneous and evoked activity. In the method of trial-to-trial variability, a negative correlation results in a reduction of the trial-to-trial standard deviation.

The relationship between spontaneous and evoked activity of the same parameter can often be summarized by a simple correlation: with a positive correlation, high prestimulus activity leads to greater evoked responses, while for a negative correlation, low prestimulus activity leads to greater evoked responses^25,26^. However, the investigation of the relationship between spontaneous and evoked activity is routinely confounded by the continued presence of spontaneous activity during the post-stimulus period; as such, when investigating dynamic relationships between spontaneous and evoked activity within one and the same variable, correlation as a method is insufficient. Two methods are presently available to assess correlations between spontaneous and evoked activity without circularity of analysis; we term these the “TTV method” and “pseudotrial method”. For the TTV method, trial-to-trial variability^25^ (TTV) is computed as the standard deviation of the signal across trials: according to the law of total variance (see Methods and Fig. 1c, right), a poststimulus reduction in TTV must be indicative of a negative correlation between spontaneous and evoked activity.

Alternatively, one can calculate the influence of prestimulus activity in a more direct way using the pseudotrial method (see Fig. 1c, left). Trials are split into groups defined by above-median and below-median pre-stimulus activity levels, and separate post-stimulus activity time courses are computed relative to these baselines. These time courses are then corrected by subtracting “pseudotrials”^26^ drawn from the intertrial interval, on which the same median-split grouping procedure has been applied (see Fig. 1c, left). This corrects for ongoing spontaneous fluctuations with the same initial conditions, and thereby corrects for regression to the mean of spontaneous activity^49^. If the trials with high prestimulus activity show a greater evoked increase (or smaller evoked decrease) than trials with low prestimulus activity, this is evidence for a positive correlation between spontaneous and evoked activity. If, by contrast, trials with high prestimulus activity show a smaller evoked increase (or greater evoked decrease) than trials with low prestimulus activity, then this is evidence for a negative correlation. As the pseudotrial method is capable of detecting both positive and negative correlations (and following our simulation results in Figs. S2 and S3), we focused our analysis on this method, using the TTV method to confirm these findings.

We applied both methods to detect the presence of a correlation between spontaneous and evoked activity in both time-domain and frequency-domain signals. To do this, we used both an MEG and an EEG dataset. The Cambridge Centre for Aging Neuroscience MEG dataset^46,47^ contains 474 subjects (in our sample) completing a brief, multisensory stimulation task: participants were asked to respond by pressing a button as soon as they see a checkerboard appear and hear a tone presented simultaneously. Our EEG dataset (*n* = 26) comes from a previous study^48^ in which participants had to choose their answers to a moral dilemma: participants had to decide whether they would push some number of people to their deaths in order to save another group. A control condition was also included where participants simply had to evaluate which group had more people. As our main questions did not relate to the particular cognitive processes involved in these tasks, both conditions were analyzed together here—they are compared in the supplementary materials.

### Spontaneous-evoked correlation in the frequency domain—effects vary by frequency band

`The first aim of our study was to investigate the relationship of spontaneous and evoked activity in multiple parameters in both the time and frequency domains. Following the observations in fMRI^25,26^, we hypothesized a negative correlation between pre- and post-stimulus activity: that is, high pre-stimulus activity should lead to a stronger decrease (or weaker increase) in post-stimulus activity than low pre-stimulus activity. Given that fMRI signals are known to be correlated with spectral power^50,51^, and following previous findings relating spontaneous and evoked alpha-band power which did not employ methods of separating spontaneous and evoked activity^32,52–54^, we hypothesized a pre-post-stimulus correlation to be visible primarily in frequency-domain representations. Our second aim was then to assess the possible differences between frequency bands in their relationship between spontaneous and evoked activity, following previous findings of differential trial-to-trial variability reduction in different frequency bands^48,55^.

**Fig. 2:**
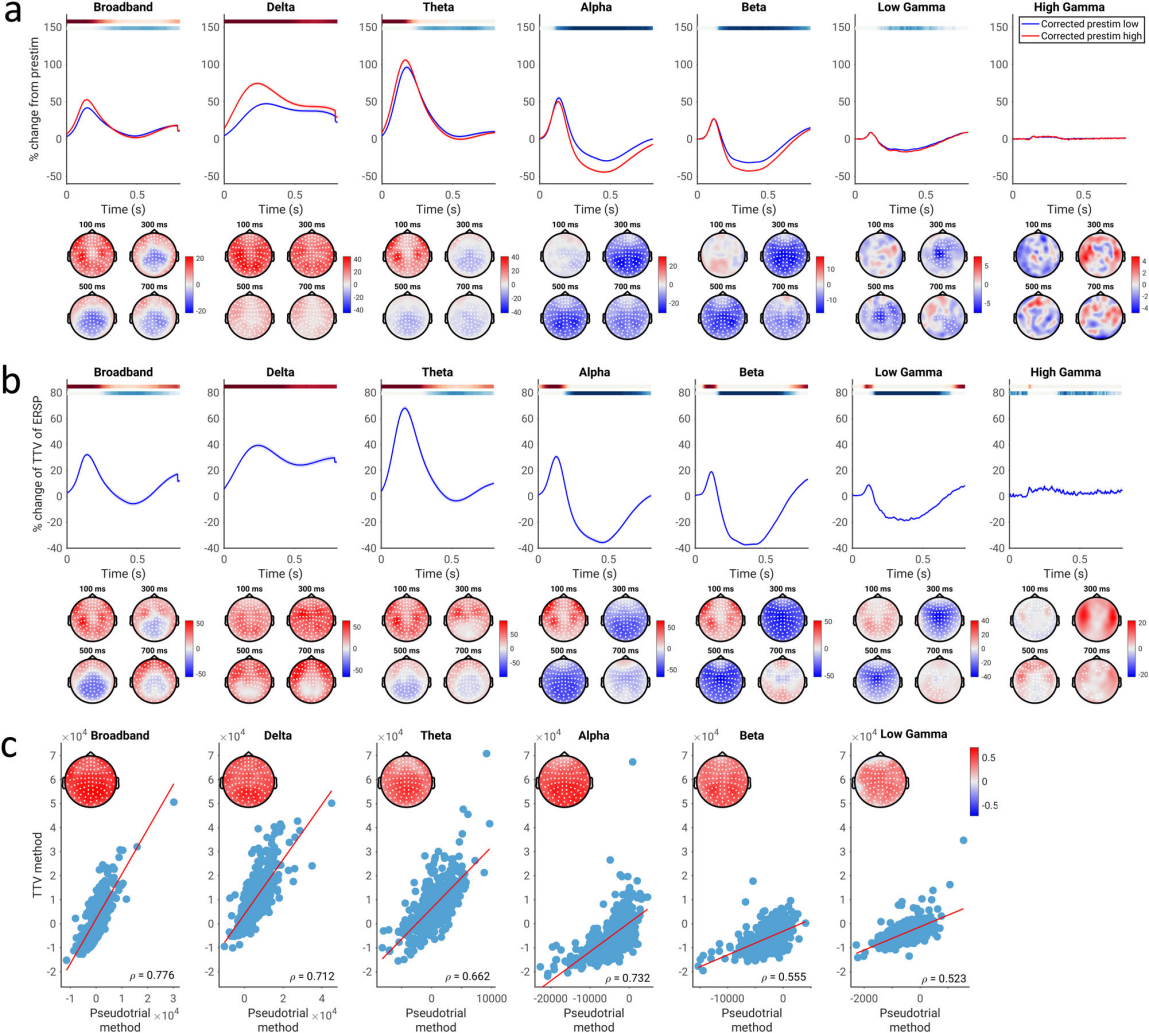
Correlation between spontaneous and evoked spectral power, assessed using the pseudotrial method and TTV method in the CamCAN dataset. **a** Pseudotrial-corrected time courses for high prestimulus (red) and low prestimulus (blue) conditions. Line shows mean across all sensors and subjects, shaded area indicates standard error of the channel-average time course across subjects. Effect topographies are shown at 100 ms, 300 ms, 500 ms, and 700 ms; white dots indicate sensors which are part of a significant cluster. Shaded overbars indicate the significance of the difference between prestim high and low conditions. The shade of the bar indicates the percent of sensors which are part of a significant cluster at any given time point (i.e., darkest = all sensors part of cluster, lightest/white = no sensors part of cluster); red indicates positive clusters (prestim high > prestim low), blue indicates negative clusters. **b** Time course of TTV in each frequency band, expressed in terms of percent change from prestimulus levels. Lines, overbars, and topographies as in a): red overbars indicate clusters with TTV increases, blue overbars indicate TTV decreases. **c** Across-subject correlation of the magnitude of spontaneous-evoked relationship found using each method. Scatter plots show the correlation of the mean values of the summary indices across all electrodes, while the topoplots show the correlations at each electrode, with white dots indicating significance following the cluster test. *N* = 474 participants.

Using the method of pseudotrials, we first tested for the presence of a correlation between spontaneous and evoked activity in MEG by comparing evoked power in trials with low vs. high pre-stimulus power (Fig. 2). We compared the pseudotrial-corrected time courses of the prestimulus high and low conditions using a Wilcoxon signed rank test at every time point and sensor; multiple comparisons were corrected for using a cluster-based permutation test over time points and sensors. Note that permutation tests are limited in the resolution of the *p*-values obtained (e.g., the minimum *p*-value for a two-tailed test with 10,000 permutations is 0.0002), so in cases where the minimum *p*-value was achieved it will be indicated as *p* ≤ 0.0002. In the broadband data, we observed a significant difference between the low prestimulus power and high prestimulus power conditions beginning at around 200 ms poststimulus (*p* ≤ 0.0002; average *d* = 0.301 for the positive cluster, average *d* = 0.245 for the negative cluster). With respect to specific frequency bands, our results show significant effects of prestimulus power in multiple bands including delta (*p* ≤ 0.0002, average *d* = 0.460), theta (*p* ≤ 0.0002 for both negative and positive clusters, average *d* = 0.313 (positive cluster) and 0.201 (negative cluster)), alpha (*p* ≤ 0.0002, average *d* = 0.379), beta (*p* ≤ 0.0002, average *d* = 0.315), and low gamma bands (*p* ≤ 0.0002, average *d* = 0.159).

We observed both negative and positive correlations between spontaneous and evoked activity in different frequency bands. Correlations were negative (i.e., high-prestimulus trials lead to lower evoked activity compared with low-prestimulus trials) in beta and especially in alpha with a 17% peak difference between the low and high prestimulus conditions, peaking between 300 and 400 ms post-stimulus. In contrast, positive correlation was found in the slower frequency bands of delta and theta, with a 33% maximum difference in delta peaking between 150 and 250 ms post-stimulus.

We next confirmed the findings of correlations between spontaneous and evoked spectral power using the method of trial-to-trial variability (TTV; Fig. 2b). We observed an early increase in TTV (between 100 and 200 ms; *p* ≤ 0.0002, average *d* = 0.432) and subsequent TTV decrease (peaking around 400 ms; *p* ≤ 0.0002, average *d* = 0.283) in broadband (Fig. 2b; *p* ≤ 0.0002). We then calculated TTV in different frequency bands. For the theta (*p* ≤ 0.0002, average *d* = 0.251), alpha (*p* ≤ 0.0002, average *d* = 0.616), beta (*p* ≤ 0.0002, average *d* = 0.961), and low gamma (*p* ≤ 0.0002, average *d* = 0.535) bands, we observed a highly significant decrease of the TTV (relative to the prestimulus period), which peaked between 400 and 500 ms. High gamma also exhibited a TTV decrease (*p* ≤ 0.0002), but the effect size was negligible (average *d* = 0.0292).

In contrast, we observed an initial increase in TTV in delta (*p* ≤ 0.0002, average *d* = 0.598) and theta (*p* ≤ 0.0002, average *d* = 0.564) bands, peaking between 150 and 250 ms. These initial increases were also observed in the alpha (*p* = 0.0008, average *d* = 0.423), beta (*p* = 0.0140, average *d* = 0.398), and low gamma bands (*p* = 0.0346, average *d* = 0.224), with these increases occurring before the subsequent TTV decreases. A significant TTV increase was also observed in high gamma (*p* = 0.0234, average *d* = 0.152), though the magnitude of this effect was very small (around 5%). Note that as discussed in the methods, the presence of a TTV increase is not indicative of a correlation between spontaneous and evoked activity in and of itself, as it may simply reflect the summation of the variances of spontaneous and evoked activity independently;^25^ however, we note that it does not contradict the results found using the pseudotrial method.

In order to validate that the two methods (pseudotrial and TTV) found similar magnitudes of the effect of spontaneous activity on evoked activity, we calculated summary indices of spontaneous-evoked relationship by taking the signed area under the curve of the TTV or prestimulus high minus low time course over each sensor and time point within the significant cluster (see Methods for details). We then correlated these summary indices across subjects at each electrode, correcting for multiple comparisons using a cluster-based permutation test (as no significant effect was observed using the pseudotrial method in high gamma, we did not correlate this effect with the TTV method). We found that the two indices were significantly correlated in all frequency bands (Fig. 2c; *p* ≤ 0.0002 in all cases, cluster-based test), suggesting that for spectral power, the two methods yield similar results.

### Spontaneous-evoked correlation in the time domain—conflict between methodologies

**Fig. 3:**
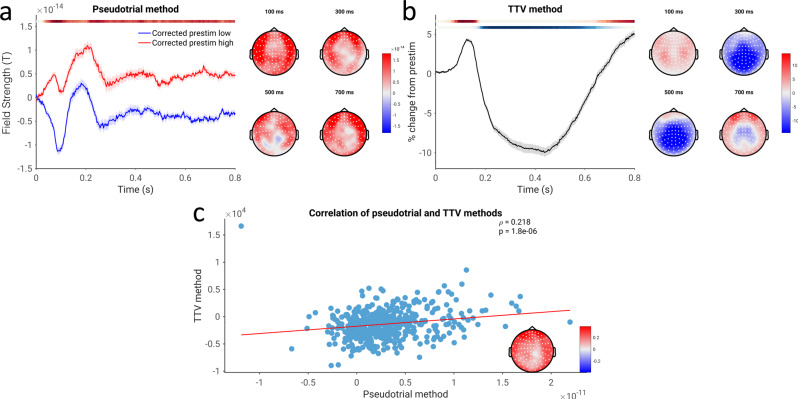
Time-domain correlation of spontaneous and evoked magnetic field strength, assessed using both methodologies in the CamCAN dataset. **a** Pseudotrial-corrected time courses for high prestimulus (red) and low prestimulus (blue) conditions. Line shows mean across all sensors and subjects, shaded area indicates standard error of the channel-average time course across subjects. Effect topographies are shown at 100 ms, 300 ms, 500 ms, and 700 ms; white dots indicate sensors which are part of a significant cluster. Shaded overbars indicate the significance of the difference between prestim high and low conditions. The shade of the bar indicates the percent of sensors which are part of a significant cluster at any given time point (i.e., darkest = all sensors part of cluster, lightest/white = no sensors part of cluster); red indicates positive clusters (prestim high > prestim low), blue indicates negative clusters. **b** Lines, overbars, and topographies as in **a**: red overbars indicate clusters with TTV increases, blue overbars indicate TTV decreases. **c** Correlation between the methodologies. Channel-average values of the summary index are plotted in the scatterplot, and the correlation coefficient at each electrode is plotted in the inset. White dots indicate sensors significant after cluster correction. *N* = 474 participants.

We next tested for the possibility of a non-additive relationship between prestimulus and post-stimulus activity in the time domain, employing the pseudotrial and TTV methods in the same way as for spectral power (Fig. 3). Using the pseudotrial method, we observed a significant positive correlation between spontaneous and evoked activity in the time domain electrophysiological signal, indicated by more positive pseudotrial-corrected post-stimulus magnetic fields for trials with high (positive) prestimulus field strength than low (Fig. 3a; *p* ≤ 0.0002, average *d* = 0.178). Interestingly, this effect was largely constant throughout the post-stimulus period, rather than peaking and decreasing as with the power-based results. In contradiction to this finding, however, we observed (as seen in previous studies^48,55,56^) a reduction in TTV, occurring from 200 ms to 700 ms post-stimulus (*p* ≤ 0.0002, average *d* = 0.528), as well as an early increase in TTV (*p* = 0.0058, average *d* = 0.328). This reduction in TTV, interpreted traditionally as in^25^, should imply a negative correlation, in direct conflict with the results observed using the method of pseudotrials. These effects were significantly correlated (*p* ≤ 0.0002), indicating that less TTV reduction implies greater positive nonadditivity. However, the magnitude of the correlation was small (ρ of channel-average summary indices = 0.218).

**Fig. 4:**
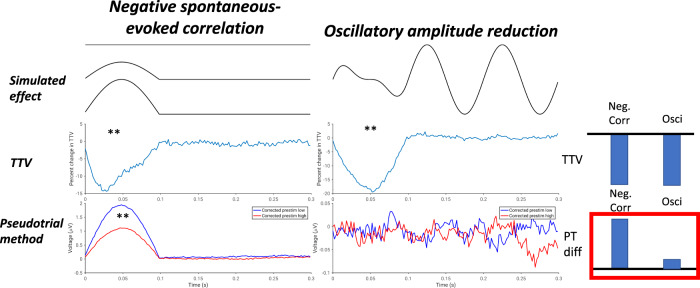
Results of simulation to disentangle methodological inconsistencies of Fig. 3. In each column, a schematic of the simulated effect is plotted (“simulated effect”), along with the normalized trial-to-trial SD (“TTV”), and the results following application of the method of pseudotrials (“pseudotrial method”). *p < 0.05, **p < 0.01. Bars at the right show a schematic of the results: both simulations show a decrease in TTV, but only the simulation with a genuine correlation between spontaneous and evoked activity shows a difference with the method of pseudotrials. *N* = 48 simulated participants.

To examine possible sources of the discrepancy between the two methods, we conducted a simulation (Fig. 4). As described in greater detail in the Materials and Methods, we simulated both a negative correlation between spontaneous and evoked voltages with no change in oscillatory power, as well as an oscillatory power reduction with no correlation between prestimulus and poststimulus voltage. We found that in both simulations, TTV decreased significantly (*p* ≤ 0.002 in each case). However, only in the spontaneous-evoked correlation simulation did we observe a significant difference between pseudotrial-corrected prestimulus high and low using the pseudotrial method (*p* ≤ 0.002). This suggests that the above findings of negative correlation using the TTV method may be confounded by the reduction of alpha power which also occurs following stimulus onset; for this reason, we view the results obtained using the method of pseudotrials as reflective of the genuine results in the time-domain signal. However, the method of pseudotrials may also be affected by the broadband nature of the ERP signal—this possibility is investigated in Fig. S14 and is elaborated on in the Discussion.

### Spontaneous-evoked correlation in the frequency domain - oscillatory and fractal components

The third aim of our study was to investigate the relationship between spontaneous and evoked activity separately in both oscillatory and arrhythmic, scale-free processes; further, we wished to examine which of these parameters accounted best for the effects observed in the mixed data. In order to examine more clearly the physiological substrates of prestimulus-dependent activity, we used the IRASA method^57^ to separate our data into oscillatory and scale-free (or “fractal”) components. We report abbreviated results of the application of this method to poststimulus activity in the Supplementary Materials (Fig. S1) as the IRASA method has never previously been applied to stimulus-locked activity; further detail on these results will be presented in a forthcoming publication. Following the results of Fig. 4, we focused our analysis of oscillatory and fractal power on the method of pseudotrials—results using the method of TTV are reported in the Supplementary Materials, and generally agree with the findings using pseudotrials (Fig. S10).

**Fig. 5:**
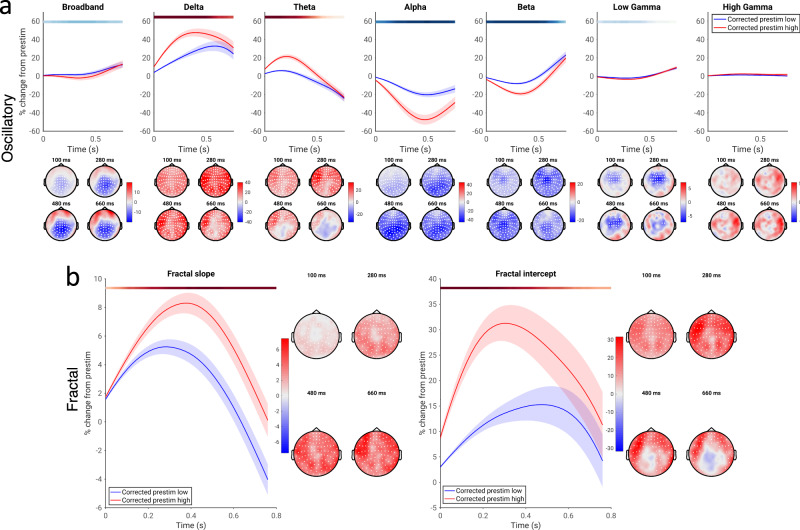
Relationship of spontaneous and evoked activity for oscillatory and fractal components of the power spectrum, assessed using the method of pseudotrials. **a** Pseudotrial-corrected time courses for oscillatory power in high prestimulus (red) and low prestimulus (blue) conditions. Line shows mean across all sensors and subjects, shaded area indicates standard error of the channel-average time course across subjects. Effect topographies are shown at 100 ms, 300 ms, 500 ms, and 700 ms; white dots indicate sensors which are part of a significant cluster. Shaded overbars indicate the significance of the difference between prestim high and low conditions. The shade of the bar indicates the percent of sensors which are part of a significant cluster at any given time point (i.e., darkest = all sensors part of cluster, lightest/white = no sensors part of cluster); red indicates positive clusters (prestim high > prestim low), blue indicates negative clusters. **b** As **a**, but for two parameters of scale-free activity: the slope (i.e., scaling exponent) and intercept (i.e., broadband offset). *N* = 49 participants.

The findings using the method of pseudotrials showed a positive correlation between spontaneous and evoked oscillatory activity in delta and theta (Fig. 5a; *p* ≤ 0.0002, average *d* = 0.812 and 0.681, respectively), as well as a negative correlation in alpha, beta, and low gamma (*p* ≤ 0.0002 alpha and beta, *p* = 0.0004 low gamma; average *d* = 0.763, 0.692, and 0.485, respectively); these results resemble the results obtained earlier when considering “mixed” power, where cortical oscillations and scale-free activity were not disentangled, suggesting that stimulus-related changes in scale-free activity did not strongly bias our previous results. Further, we quantitatively compared the magnitudes of spontaneous-evoked correlation between oscillatory and fractal activity, finding that oscillatory activity within a given frequency band generally displayed stronger correlations between spontaneous and evoked dynamics than fractal power within the same frequency range (Fig. S11).

Scale-free activity can be described by activity which follows a power-law distribution, with power distributed as 1/f^β^: this can be modeled by a linear fit on a log-log scale. Scale-free activity can thus be described by two parameters: the scaling exponent, which reflects the slope of this linear fit (the β parameter in the aforementioned distribution), and the broadband offset, which reflects the y-intercept of the fit (though see the Discussion on the issue of multifractal dynamics, which are not considered here). We therefore examined whether these two parameters associated with scale-free activity, the scaling exponent and broadband offset, exhibited correlations between their spontaneous and evoked dynamics. We found that both the slope (scaling exponent) and intercept (broadband offset) of fractal activity exhibited a positive correlation between their spontaneous and evoked activity (Fig. 5b): high values of each parameter in the prestimulus period led to greater stimulus-evoked increases of each parameter (*p* ≤ 0.0002 in both cases; average *d* = 0.651 for slope, 0.679 for intercept). However, we also note a negative correlation between spontaneous and evoked dynamics of the broadband offset over central sensors, which reached significance using the method of TTV (see Fig. S10), but not the method of pseudotrials. Together, our results suggest distinct contributions of oscillatory and arrhythmic/fractal components to positive and negative correlation schemes in spontaneous and evoked activity, and that the correlations observed in the mixed data are largely attributable to cortical oscillations, rather than scale-free activity.

### Replication of spontaneous-evoked correlation in an independent EEG dataset

**Fig. 6:**
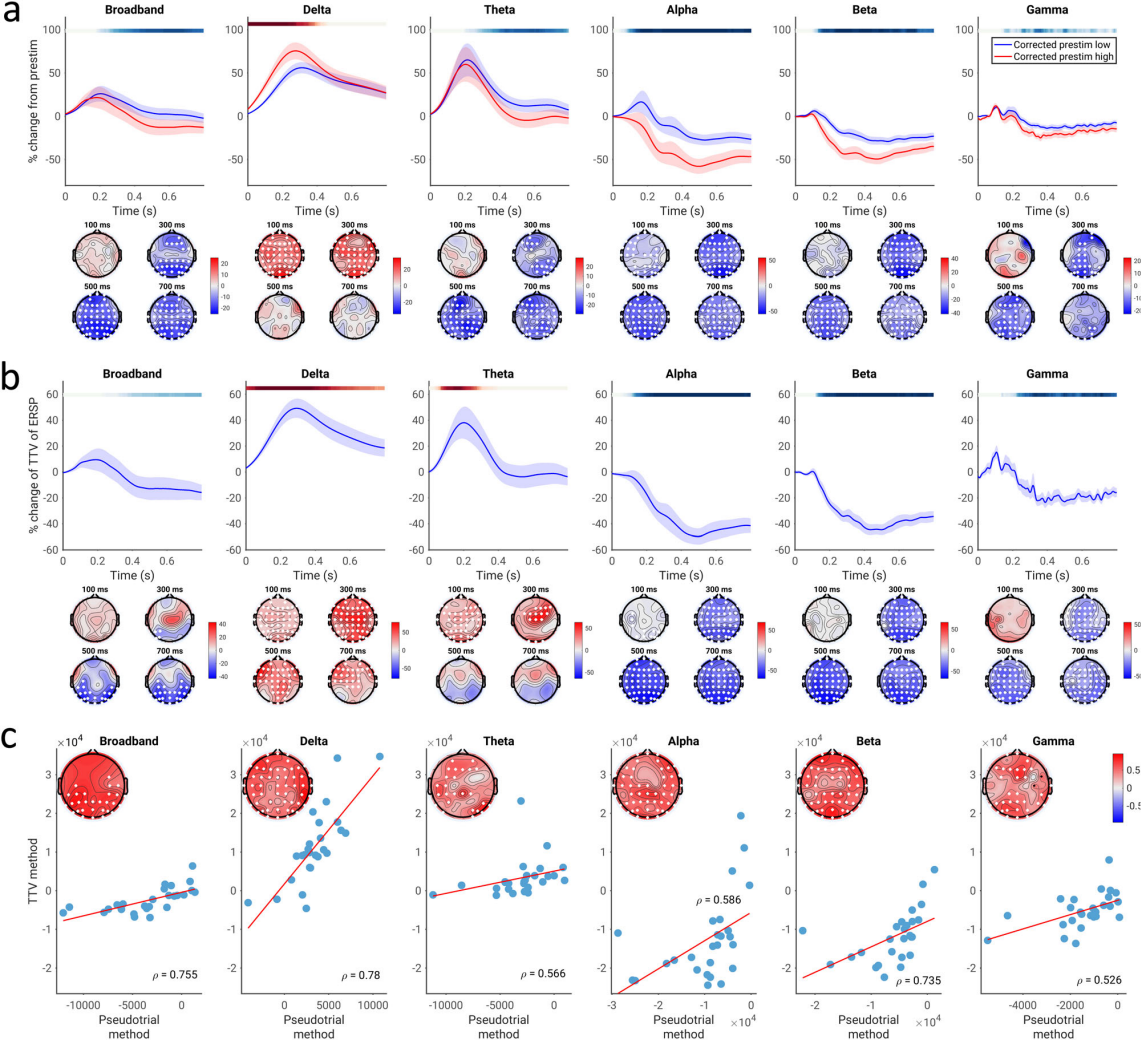
Correlation of spontaneous and evoked spectral power in the replication EEG dataset (equivalent to Fig. 2 for the main dataset). **a** Pseudotrial-corrected time courses for high prestimulus (red) and low prestimulus (blue) conditions. Line shows mean across all sensors and subjects, shaded area indicates standard error of the channel-average time course across subjects. Effect topographies are shown at 100 ms, 300 ms, 500 ms, and 700 ms; white dots indicate sensors which are part of a significant cluster. Shaded overbars indicate the significance of the difference between prestim high and low conditions. The shade of the bar indicates the percent of sensors which are part of a significant cluster at any given time point (i.e., darkest = all sensors part of cluster, lightest/white = no sensors part of cluster); red indicates positive clusters (prestim high > prestim low), blue indicates negative clusters. **b** Time course of TTV in each frequency band, expressed in terms of percent change from prestimulus levels. Lines, overbars, and topographies as in **a**: red overbars indicate clusters with TTV increases, blue overbars indicate TTV decreases. **c** Across-subject correlation of the two methods. Scatter plots show the correlation of the mean values of the summary indices across all electrodes, while the topoplots show the correlations at each electrode, with white dots indicating significance following the cluster test. *N* = 22 participants.

**Fig. 7:**
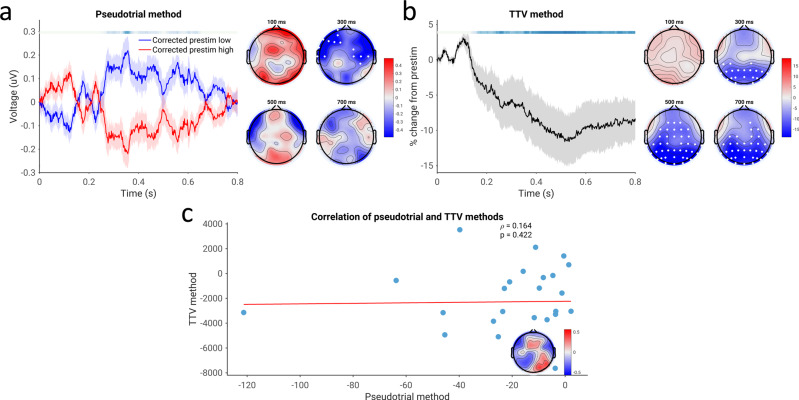
Correlation of spontaneous and evoked EEG voltage for the replication EEG dataset (equivalent to Fig. 3 in the main dataset). **a** Pseudotrial-corrected time courses for high prestimulus (red) and low prestimulus (blue) conditions. Line shows mean across all sensors and subjects, shaded area indicates standard error of the channel-average time course across subjects. Effect topographies are shown at 100 ms, 300 ms, 500 ms, and 700 ms; white dots indicate sensors which are part of a significant cluster. Shaded overbars indicate the significance of the difference between prestim high and low conditions. The shade of the bar indicates the percent of sensors which are part of a significant cluster at any given time point (i.e., darkest = all sensors part of cluster, lightest/white = no sensors part of cluster); red indicates positive clusters (prestim high > prestim low), blue indicates negative clusters. **b** Time course of trial-to-trial variability. Lines, overbars, and topographies as in a): red overbars indicate clusters with TTV increases, blue overbars indicate TTV decreases. **c** Correlation of the two methods. Mean values of the summary indices across sensors are plotted in the scatterplot, and the correlation coefficient at each electrode is plotted in the inset. White dots indicate sensors significant after cluster correction. *N* = 22 participants.

To ensure robustness of the findings, we replicated our procedure in an independent EEG dataset (Figs. 6 and 7). This dataset consisted of a more cognitively demanding paradigm, a moral decision-making task previously described in Wolff et al^48^. in which participants decide whether they are willing to sacrifice a group of people to save another. Using the method of pseudotrials, we generally found the same pattern of spontaneous-evoked correlation as in Fig. 2, with delta displaying a positive correlation and alpha, beta, and gamma showing negative correlation (Fig. 6a; *p* ≤ 0.0002 for each; delta: average *d* = 0.787; alpha: average *d* = 0.884; beta: average *d* = 0.743; gamma: average *d* = 0.543). In contrast, however, we observed a negative correlation in the theta band in the EEG data (*p* ≤ 0.0002; average *d* = 0.632), where we had observed a positive correlation in MEG. We submit that this difference in theta may be due to the band being a transition between the positive correlation regime in delta and the negative one in alpha and beta. In the EEG data, the evoked delta is smaller relative to the evoked alpha than in the MEG data; regardless of whether this is due to task effects or the difference in imaging modality, this difference in relative contribution may explain the different correlation scheme in theta.

The results using the method of trial-to-trial variability largely confirm the results obtained using the method of pseudotrials (Fig. 6b; *p* = 0.0274, average *d* = 0.691 for broadband; *p* = 0.0174, average *d* = 0.564 for theta, *p* ≤ 0.0002 for each other band; average *d* = 0.811 for delta, 1.01 for alpha, 1.35 for beta, 0.764 for gamma). The only exception is that the TTV method does not indicate a negative correlation in the theta band—however, as mentioned by He^25^ and in the introduction, this does not mean that such a correlation is definitively not present, only that in this case the variability of the spontaneous and evoked activity is sufficient to overwhelm the term related to their correlation. For all bands, the effect magnitudes calculated with each method were correlated (Fig. 6c; *p* = 0.0004 for alpha, *p* ≤ 0.0002 for each otherband).

We also assessed spontaneous-evoked correlation in the time domain in EEG data. Similar to the MEG data, we observed a decrease in TTV over much of the poststimulus period (Fig. 7b; *p* = 0.0008, average *d* = 0.704). However, in contrast to the MEG findings, we observed a negative correlation between spontaneous and evoked activity in the time-domain EEG data using the method of pseudotrials (Fig. 7a; *p* = 0.0062, average *d* = 0.509). Results from the method of pseudotrials and the TTV method were uncorrelated (Figure 8c; *p* = 0.240). However, we note that when accounting for filtering effect**s** in the MEG data, a negative correlation between spontaneous and evoked activity emerges over central sensors, corroborating the EEG findings using the method of pseudotrials—see the discussion and Fig. S12 for further treatment of this difference from the MEG data.

### Control analyses and simulations

We additionally ensured that the length of the prestimulus period did not affect the findings. In addition to the presently used 100 millisecond window, a 50-ms prestimulus period (Figs. S4–S5) and a 200-ms prestimulus period (Figs. S6–S7) were used. We further attempted to control for anticipation effects by taking only the longer ITIs and placing pseudotrials randomly within these long ITIs (Figs. S8 and S9). These values had little discernable effect on the main results, though the time-domain data appeared sensitive to anticipation effects.

In order to distinguish true correlations between spontaneous and evoked activity from potentially spurious correlations due to trial-by-trial variations in noise, we employed two simulations: one with a purely additive relationship between spontaneous and evoked activity (additive simulation; Fig. S2), and another in which a negative correlation was present (non-additive simulation; Fig. S3). In the additive simulation, no evidence of spontaneous-evoked correlation was observed using the pseudotrial method. However, several significant decreases of TTV were observed, as well as increases in different noise conditions. In contrast, in the non-additive simulation, we observed consistent negative correlation using the method of pseudotrials and the method of TTV in the low-noise conditions. This confirms that the method of pseudotrials is capable of detecting true correlations between spontaneous and evoked activity given a sufficient signal-to-noise ratio, and does not suffer from a high degree of false positives due to time-varying noise. The method of TTV also appears capable of detecting such correlations, but in certain noise regimes it can be subject to false positives.

We also attempted to control for the potential inclusion of part of the post-stimulus response in the pre-stimulus period. This could occur for a variety of reasons, including high-pass filtering in the case of the time-domain signal (Fig. S12), and because of the inherent trade-off between time and frequency resolution in the case of frequency-domain signals (Fig. S13). We found that while these factors may have resulted in overestimation of the magnitude of positive correlations in our data, these positive correlations remained significant when controlling for them.

### Behavioral analyses

While the primary focus of our paper was on the relationship between spontaneous and evoked neural activity, we also investigated behavioral relationships with the available data we had. First, we related prestimulus and poststimulus spectral power changes with reaction times in the CamCAN dataset using mixed-effects models, confirming previously-reported relationships in a larger sample (Fig. S16). Secondly, we assessed whether the different task conditions in the EEG dataset led to different profiles of spontaneous-evoked correlation (Fig. S17): we found that spectral power (with the minor exception of gamma-band power) showed similar patterns of spontaneous-evoked correlation in both task conditions, further supporting the assumption that relationships between spontaneous and evoked activity in spectral power are task-general.

## Discussion

The goal of our study was to examine the dynamics of the relationship between spontaneous and evoked activity in multiple electrophysiological parameters. For this purpose we used robust methods which controlled for the carry-over of spontaneous activity into evoked activity to assess their correlation. We show strong and distinct relationships of spontaneous activity in the prestimulus period with evoked activity in the poststimulus period in several neurophysiological variables. The observed correlation was negative in the alpha and beta bands (such that high alpha/beta amplitude pre-stimulus leads to greater stimulus-evoked desynchronization), and positive in the delta band (such that high delta amplitude pre-stimulus leads to greater stimulus evoked synchronization). Moreover, we demonstrate that this effect is primarily found in band-limited oscillatory dynamics, rather than aperiodic, scale-free dynamics, though scale-free activity also displays a relationship between spontaneous and evoked dynamics of the scaling exponent and broadband offset. Finally, we also observed spontaneous-evoked correlation in the time domain, with a positive correlation between spontaneous and evoked activity observed in MEG in the multisensory task, and a negative correlation in the EEG moral reasoning task.

These results show that for a variety of neurophysiological parameters, trial-by-trial fluctuations in the pre-stimulus period influence variation in post-stimulus neural responses to stimuli in predictable ways. Our results extend recent findings relating prestimulus spectral power to ERP components^27,52–54,58–61^, and complement cellular and modeling research investigating this question^36,62–64^. While negative correlation between spontaneous and evoked alpha band power has been suggested by^52–54^, our study is the first to provide robust evidence of this phenomenon using (a) statistics which avoid circular analysis, (b) multiple imaging modalities, and (c) a very large sample size. Further, we show for the first time differential relationships between spontaneous and evoked activity in different electrophysiological variables, including differences between frequency bands, between oscillatory and scale-free processes, and between time-domain and frequency-domain electrophysiological signals. In future, such research may contribute to a more complete understanding of how fluctuations in spontaneous activity mediate variability in behavioral and cognitive features like perception, self, attention, and consciousness.

Previous studies examining the relationship of spontaneous and evoked activity in EEG have generally started from a physiological background, assessing the influence of general states of arousal and desynchronization on evoked responses. These studies usually assess different electrophysiological features in the prestimulus and poststimulus periods, rather than examining the same variable in both periods^27–29,31,33^. While this approach is useful in demonstrating a link in principle between spontaneous and evoked activity, it may miss subtler relationships present in the dynamics of particular electrophysiological variables which may be important to understanding how spontaneous pre-stimulus activity shapes stimulus-evoked activity, or important to applications or analyses which center on specific frequency bands or electrophysiological quantities. Moreover, the characterization of pre- and post-stimulus periods by distinct measures does not necessarily avoid the methodological challenges of assessing the relationship between spontaneous and evoked activity, as EEG metrics are highly interrelated and confounded with one another: ERP components are related to power and phase changes of cortical oscillations^52,65^, entropy measures (such as those used by^33^ to assess desynchronization) are related to spectral power^66^, and, as previously mentioned, cortical oscillations and scale-free activity are frequently confused for one another^57,67^. Given this, correlations between seemingly different measures in the pre- and post-stimulus periods may lead to spurious effects due to the non-independence of the measurements in the pre- and post-stimulus period. Using methods developed in fMRI to account for the continued presence of spontaneous activity in the poststimulus period, our paper addresses the correlation between spontaneous and evoked activity in the dynamics of distinct electrophysiological variables for the first time in EEG. Our results confirm relationships between spontaneous and evoked activity and extend this previous work by showing the specific relationships between ongoing and evoked activity of different electrophysiological variables of interest.

Our finding of widespread non-additive relationships between pre- and post-stimulus activity of the same parameters carries implications for the analysis of evoked activity in EEG/MEG, where trial-based paradigms which assume linear superposition of spontaneous and evoked activity are the dominant way of conducting task-related neuroscience research^68^. Our results join numerous others^25,27,29,33^ to suggest that by ignoring the impact of prestimulus activity, this approach misses important data related to the dependence of the evoked response on pre-stimulus and ongoing activity (as noted previously in fMRI by^25^). For example, averaging all trials may lead one to conclude that an evoked response is small or zero, when in reality there are opposite modulations depending on prestimulus activity; alternatively, interpretational difficulties may emerge if condition differences in evoked activity are instead due to condition differences in spontaneous activity. Our data, emphasizing the impact of continuous fluctuations in spontaneous activity on transient stimulus-evoked responses, provide support for recent attempts to develop analysis strategies for continuous paradigms which do not require the averaging of multiple trials^68^, and encourages efforts to translate non-additive models of the relationship of spontaneous and evoked activity^69–71^ into flexible experimental designs and analysis strategies.

### Distinct relationships of spontaneous and evoked activity in different electrophysiological variables - positive and negative correlation in different frequency bands

We demonstrate a non-additive relationship between prestimulus and poststimulus activity in multiple electrophysiological variables. For spectral power, we show similar patterns of correlation between spontaneous and evoked activity in different paradigms and modalities, i.e., sensory in MEG and cognitive in EEG, as well as using different methods (TTV^25^ and pseudotrial^26,48^). This suggests that the close relationship between spontaneous and evoked activity in spectral power is a robust phenomenon that holds across different tasks and methods. Most interestingly, we demonstrate two different correlation schemes holding in the spectral power of different frequency bands. Negative correlation between spontaneous and evoked activity is predominant in alpha in the later post-stimulus periods (around 400 ms). In contrast with these results and with previous reports in fMRI, positive correlation is observed in the delta band, occurring earlier around 200 ms.

The existence of a negative correlation between spontaneous and evoked alpha power is noteworthy given prior data showing the impact of pre-stimulus alpha on post-stimulus perception^14,17^, self^15^, and conscious awareness of stimuli^18,72^. Prestimulus alpha has also been well-studied for its influence on post-stimulus ERPs^28,54,61^, with prestimulus alpha power being predictive of the P1 and N1 ERPs in particular^27,58^. Alpha has traditionally been regarded as an inhibitory process, with alpha desynchronization reflecting release from inhibition; according to Klimesch^73^, this reflects controlled access to the “knowledge system”. Furthermore, prestimulus or anticipatory alpha desynchronization has been associated with attention allocation and better subsequent performance on perceptual tasks^74^. Given these observations, the here observed negative correlation of spontaneous and evoked alpha power may serve a cognitive purpose, though the exact nature of this purpose remains unclear.

Additionally, we observed a positive correlation between spontaneous and evoked activity in the delta band. Delta-band activity has been shown to be involved in arousal and attention in the waking state^75,76^, including mediating the p300 event-related potential^65^. It is also known as an electrophysiological correlate of activity in the default-mode network^77^, and as a modular of motivation, arousal and homeostasis^78^. Prestimulus delta-band power has been shown to be predictive of the P3 ERP component, which itself is thought to reflect delta phase synchronization;^79,80^ our results extend this finding by showing that this effect is true for delta power as well as phase, and is not influenced by the carry-over of spontaneous activity into the poststimulus period. Though our findings leave open the exact function of positive correlation between spontaneous and evoked delta power, it may provide a mechanism linking motivation and arousal with responses to salient stimuli. However, we note that the magnitude of the positive correlation in delta may be overestimated due to the temporal imprecision associated with estimating slow-frequency activity (see Fig. S14), and urge caution in the interpretation of these results.

Interestingly, the consistency of our spectral power results between different modalities and paradigms differs from previous findings, which showed task-specific relationships between pre-stimulus spectral power and poststimulus ERP components^29,79,81^. This may, in part, be due to the fact that we investigated the same neurophysiological variables during the transition from prestimulus to post-stimulus period; alternatively, they may be due to the use of time-domain signals to operationalize stimulus response, as we likewise observed task-specific spontaneous-evoked correlations in our ERP data. These findings encourage future research on the question of the mechanisms underlying such task-general relationships between spontaneous and evoked activity, building on the accounts in^79^ for positive correlation and the models of^70,82^ for negative correlation.

### Spontaneous-evoked correlation in different electrophysiological variables—time vs. frequency domains, oscillatory vs. fractal dynamics

Using the method of pseudotrials, we observed a correlation between spontaneous and evoked activity in the time-domain electrophysiological signal of both modalities; however, we found differences in these correlations between the MEG and EEG datasets. In the MEG dataset, we observed a positive correlation between spontaneous and evoked magnetic field strength over most of the prestimulus period, while in EEG we observed a negative correlation in the late poststimulus period (300 ms to 600 ms). This may reflect a difference between the two modalities, as MEG and EEG are sensitive to different cortical sources^83^. Alternatively, it could be that the task (simple sensory vs. complex cognitive) may have an effect on the correlation scheme exhibited by the time-domain signal. We note in particular that when an alternative high-pass filtering scheme is used in the MEG data (using only the hardware filter at 0.03 Hz rather than implementing Butterworth high-pass filtering at 1 Hz, as done in the main text), these results appear more similar: the positive correlation originally observed is now limited to the first 200 milliseconds, and the same negative correlation observed in EEG now re-emerges. However, it may also be the case that the whole poststimulus-period positive correlation is not observed in EEG due to the effects of the reference: MEG signals, being reference free, may be able to detect this effect.

Our results suggest that several common ERP components may exhibit dependence on spontaneous activity. In particular, our MEG results suggest that at the very least the early components, i.e. N1 and P1, exhibit positive correlations with ongoing activity, such that when voltage is high and membranes are strongly depolarized, P1 amplitudes are greater and N1 amplitudes are more positive (i.e., lower). This joins previous research which has investigated the impact of ongoing spectral dynamics on N1 and P1 amplitudes^27,58,59^. Further, our EEG results (and our MEG results when high-pass filtering is removed) both suggest the presence of a negative correlation between ongoing activity and amplitudes of the late positive potential; this is consistent with the non-zero-mean oscillation framework of^52^. However, because of the methodological issues pointed out in Fig. 4 and Fig. S12, more research is needed to investigate spontaneous-evoked correlation in time-domain electrophysiological signals and the conditions that affect it.

Finally, we observed differential relationships between spontaneous and evoked activity in scale-free dynamics and cortical oscillations. This complements recent observations of the differential relationships of oscillatory and scale-free dynamics to the fMRI signal^50^, differential contributions to cognitive processing speed^84^, and differential modulation by psychedelic drugs^85^. The patterns of correlation between spontaneous activity and evoked activity observed in the mixed power were highly similar to those observed in cortical oscillations; further, we observed a larger influence of spontaneous activity on evoked responses in the oscillatory rather than the scale-free component of the power spectrum, reinforcing the central role of cortical oscillations in stimulus response and suggesting that at least in our case, scale-free activity did not exert a substantial bias on our mixed-power results. Interestingly, we also found correlations between spontaneous and evoked dynamics of both parameters describing scale-free activity, the scaling exponent and broadband offset: each of these displayed a positive correlation between their spontaneous dynamics and evoked amplitudes. Given that the scaling exponent of fractal activity has previously been related to excitation-inhibition balance^42,43^, this raises intriguing questions for future research about the dynamics of this balance and how it is modulated by external perturbations.

### Limitations

As discussed above, a central limitation of our study (and others investigating the same question) is the methodological difficulty of assessing correlations between spontaneous and evoked activity when activity in the post-stimulus period reflects a mixture of the two. While unlike previous studies in electrophysiology, we employed methods to account for this issue, two main challenges emerged in our study: the failure of the TTV method in time-domain electrophysiological data, and the confounding of positive correlation estimates by temporal imprecision. As shown in Fig. 4, TTV decreases in the raw electrophysiological signal can be induced by desynchronization of oscillations, precluding their interpretation as being indicative of a correlation between spontaneous and evoked activity.

Alteratively, it may be the case that the pseudotrial method is not suited for investigating broadband, time-domain electrophysiological data. Since M/EEG data contains fluctuations in multiple frequencies, it may be that a single prestimulus window of fixed duration does not capture the “up and down” states of different physiological processes which fluctuate at different time scales, and as such the method of pseudotrials may fail (e.g., 100 ms, the prestimulus length employed in our study, may be too long to capture meaningful variation in spontaneous beta or gamma power, for instance). We attempted to address this possibility in the supplementary materials (Fig. S14) by filtering the time-domain data into frequency bands and assessing spontaneous-evoked correlation in each band with a band-dependent prestimulus window. While this procedure improved the correspondence between the TTV and pseudotrial methods in some cases (e.g. in the delta and alpha bands), notable discrepancies remained (e.g., the beta band, where the two methods again yielded opposite results). These data also yielded some support for the assumption of non-zero mean oscillations^52^, which may drive some part of the time-domain correlations: however, since the filtering of time domain data to analyze stimulus evoked changes in different bands is a fairly uncommon procedure, more research is needed to investigate this and interpret these findings in terms of traditional measures such as ERP components, which we focused our time-domain analyses on, or in terms of phase synchronization/shifting of oscillations.

In the case of positive correlations between spontaneous and evoked activity, the method of pseudotrials can also be affected by any mechanism which “smears” poststimulus activity back into the prestimulus period, such as highpass filtering in the time domain (Fig. S13), or the inherent imprecision in estimating low-frequency power (Fig. S14). While all our results remain significant while considering these effects, this issue does appear to result in overestimation of the magnitude or temporal extent of positive correlation, and as such more research is needed to confirm the positive correlations between spontaneous and evoked low-frequency power, time domain signals, and scale-free parameters seen in our data. In particular, methods which focus on inter-subject consistency of responses in order to isolate task-evoked activity (such as the one applied by Lynch et al.^86^) may be useful in these cases, but they have not yet been developed for the purpose of relating spontaneous and evoked activity. Alternatively, single-trial modeling procedures may be developed based around the same principle as the method of pseudotrials (i.e., controlling for the autocorrelation of spontaneous activity) by, for instance, rank-ordering pseudotrials and real trials based on their prestimulus activity, and treating the pseudotrials as covariates—we encourage future researchers to take up this challenge.

We were not able to determine a particular mechanism which explained the correlation patterns we observed. In a preliminary investigation, we found no influence of phase coherence or phase-amplitude coupling (Fig. S15) on the negative correlation scheme observed in our study between spontaneous and evoked high-frequency power. However, we did not investigate these possibilities in detail; further empirical and modeling work is necessary to determine more precisely why and how spontaneous and evoked activity interact in the ways we observed.

Finally, our investigation of spontaneous-evoked correlation in scale-free activity was not able to address the issue of multifractal dynamics. Recently, the possibility that multiple scaling regimes exist in the brain has been raised by numerous publications^67,87,88^, suggesting that a simple linear relationship between log power and log frequency is insufficient to capture scale-free dynamics in the brain. Unfortunately, the IRASA method which we employed is ill-suited to examine different scaling regimes, as the resampling procedure it employs blurs the boundaries between them^57,67^. However, parametrization methods which have recently been employed to estimate these “knee” frequencies face challenges when operating on single-trial power spectra;^67^ in contrast, the IRASA method, by estimating the fractal power spectrum as a median of multiple resampled spectra, is robust to trial-level noise, making it the ideal candidate for our purposes despite this limitation. Future work should further explore the stimulus-evoked dynamics of multifractal activity, including the relationship between its spontaneous and evoked dynamics.

## Conclusion

In this paper, we investigated the relationship between spontaneous pre-stimulus and stimulus-evoked activity in different electrophysiological variables. Using both MEG and EEG and applying robust analysis methods, we observed, as hypothesized, that multiple electrophysiological variables exhibited distinct relationships between their spontaneous activity in the pre-stimulus period and their post-stimulus evoked activity. Positive correlation between spontaneous and evoked activity was found in delta power, while negative correlation occurred in alpha and beta dynamics. Both forms of spontaneous-evoked correlation were found robustly in the dynamics of spectral power, and predominantly in oscillatory rather than arrhythmic/scale-free dynamics. Analogously, positive and negative correlations could also be observed in the time-domain electrophysiological signal.

This work carries methodological implications for our understanding of stimulus-induced or task-evoked activity by unraveling, in part, the mechanisms by which it is shaped by spontaneous neural activity. More importantly, these findings in future may provide novel insight into the neuronal mechanisms of perception, cognition, and consciousness, as these phenomena are influenced by the relationship between spontaneous and evoked activity.

## Materials and methods

### Methodologies for assessing correlations between spontaneous and evoked activity

fMRI studies investigating the relationship of prestimulus and poststimulus activity have employed two distinct methods: trial-to-trial variability (TTV)^25^ and pseudotrials^26^. The method of *trial-to-trial variability* (TTV) makes use of the law of total variance in assessing a correlative relationship between spontaneous activity (X) and evoked activity (Y):

1 $${\sigma }_{X+Y}^{2}={\sigma }_{X}^{2}+{\sigma }_{Y}^{2}+2{r}_{{XY}}{\sigma }_{X}{\sigma }_{Y}$$

A putative correlation between spontaneous and evoked activity is represented by the correlation *r*_*XY*_. Since variances are always positive, the only circumstance in which one could observe a reduction in trial-to-trial variability is if this coefficient is negative—hence, a reduction in TTV implies a negative correlation between spontaneous and evoked activity. However, an increase in TTV could be produced by any of the three models. It was found that the trial-to-trial variability of the fMRI response decreased following stimulus onset, a pattern which could only be explained assuming a negative correlation between spontaneous BOLD dynamics and the evoked BOLD signal. This phenomenon has frequently been associated with attractor models of the brain^89^.

More recently, a more direct method has emerged to assess the presence of correlations between spontaneous and evoked activity in fMRI^26^. An intuitive way to assess this relationship would be to compare the time courses of trials with high and low prestimulus activity, for instance by splitting trials based on the median prestimulus values. However, such an approach can be confounded by regression to the mean^49^—trials which are selected based on having high prestimulus activity may naturally return to a more average value, simply because they are selected as having “above average” values in the first place, or by other dynamical features of the spontaneous activity. To correct for this, Huang et al^26^. applied the same procedure to “pseudotrials”, segments of the task recording where no stimulus is present. Pseudotrials are split into groups based on the median of their own “prestimulus” activity (activity before pseudotrial onset): this provides an estimate of the response of spontaneous activity to the median split procedure. Pseudotrial time courses in each prestimulus condition are then subtracted from the real trial time courses in each condition. The remaining differences between the real trial prestimulus high and low groups reflects the genuine influence of spontaneous activity on evoked activity. An advantage of this method is that it can theoretically detect a positive correlation between spontaneous and evoked activity and distinguish it from a negative one, which cannot be accomplished with the method of trial-to-trial variability. Using this method, a negative correlation was observed between the spontaneous and evoked BOLD signal, which correlated with the same effect assessed with the TTV method^26^.

### Datasets and experimental designs

We used two previously published datasets to investigate our hypotheses, in two different imaging modalities. As our main dataset, we used 474 subjects from the Cambridge Centre for Ageing and Neuroscience (CamCAN) MEG dataset^46,47^ (available at http://www.mrc-cbu.cam.ac.uk/datasets/camcan/). Data were recorded using a Vectorview 306-channel MEG system (Elekta Neuromag, Helsinki) in a light magnetically-shielded room. The task associated with this dataset was a simple sensorimotor task, in which participants were presented with a multimodal auditory (300 ms tone) and visual (34 ms checkerboard pattern) stimulus. 120 trials were presented, and the inter-trial intervals (ITIs) were jittered between 2 and 26 s. The auditory and visual stimuli were presented simultaneously, and participants were required to respond with a button press once they perceived the stimulus. Eight trials were also included of unimodal auditory or visual stimuli (four trials each)—these were also included in the analysis. Details of the task can be found in^47^.

To replicate the findings, we used data from a moral decision-making paradigm, previously published in^48^. Participants (*n* = 26) in this task were presented with a moral dilemma, in which participants would push a set of bystanders in front of a trolley in order to save another group from death (Philippa Foot’s “Footbridge dilemma”). Participants were asked to respond either yes or no to the ratio of those killed to saved (in visual stimulus) by pushing a button. An additional externally-guided condition required participants simply to compare the number of people on the right side of the screen with the left. For the purposes of our study, we considered both conditions together. A total of 420 trials were included in the analysis, and the ITI was jittered between five and six seconds. Further details can be found in Wolff et al.^48^.

### Data preprocessing

At the time of analysis, fully preprocessed data were not available from the CamCAN repository. As such, we applied standard preprocessing steps in Fieldtrip^90^ and MNE^91^ to further clean the data. Our starting point for preprocessing was the MaxFiltered data provided in the CamCAN data release (see^46^ for details of the MaxFilter steps). For ease of preprocessing and analysis, gradiometer channels were removed, and only magnetometers were analyzed. Data were first downsampled to 500 Hz, then bandpass filtered from 1 to 200 Hz with a fourth-order Butterworth filter. In order to remove high-amplitude transient artefacts which could bias independent component analysis (ICA) decomposition, Autoreject^92^ was applied to find and label data epochs with artefacts. Following the methods of the Human Connectome Project^93^, 20 ICA iterations were then performed, with artefactual epochs from the previous step excluded from the ICA training. Artefactual components were automatically labeled using in-house modifications of scripts from the Human Connectome Project’s megconnnectome software^93^ and removed from the original (pre-Autoreject) data. Data were then epoched from 2 s prior to stimulus onset to 1.5 s poststimulus, and Autoreject was run a second time to repair any trials with artefacts remaining.

Data from the moral decision-making paradigm were preprocessed in EEGLAB^94^. First, data were downsampled to 500 Hz and bandpass filtered from 1 to 50 Hz. High-amplitude artifacts were removed prior to ICA using Artefact Subspace Reconstruction^95^, and data were re-referenced to an average reference. Data were then epoched from 3 s prior to stimulus onset to 2 s poststimulus. ICA was then run on the data, and bad components were identified using an automated algorithm^96^. For details of the data collection, see Wolff et al^48^.

### Definition of real trials and pseudotrials

For each dataset, the prestimulus period for real trials was taken as the interval from 100 milliseconds before stimulus onset to stimulus onset, with the real trial poststimulus period falling from 0 to 800 milliseconds. Similarly, “pseudotrials” were defined as the period from 900 to 100 milliseconds pre-stimulus, with the period from 1000 to 900 milliseconds pre-stimulus serving as the “prestimulus” period for that pseudotrial. We tried several different lengths of the prestimulus interval, to ensure that this choice had minimal impact on the results. These findings are reported in the supplementary materials (Figs. S4–S7).

### Estimating correlations between spontaneous and evoked activity

We used both the method of pseudotrials^26^ and the method of trial-to-trial variability^25^ to assess the presence of a non-additive relationship between spontaneous and evoked activity in various signals. For each signal, we averaged the amplitude across time points in the prestimulus window to get a single value characterizing prestimulus amplitude for each subject, channel, and trial (including pseudotrials). For each subject, we then split trials and pseudotrials separately into high or low prestimulus amplitude groups based on the median of their respective prestimulus activity. For spectral power, pseudotrial and real trial time courses were then normalized by the average prestimulus value over all trials and expressed in terms of percent change from this value. This normalization was not done for time-domain signals, as the average prestimulus value was approximately zero.

We then computed the “corrected” time courses by subtracting the prestimulus-normalized real trial time courses in each bin from the prestimulus-normalized pseudotrial time courses (see ref. ^26^)—i.e., pseudotrials in the high prestim condition were subtracted from real trials in the high prestim condition, and pseudotrials in the low prestim condition were subtracted from real trials in the low prestim condition. This “corrected” time series is now controlled for regression to the mean, as this natural difference between prestim high and low is captured by the pseudotrial dynamics and removed from the data. We then tested for whether the corrected time series for high and low prestimulus amplitude were significantly different from each other using the cluster-based procedure described below.

For the method of trial-to-trial variability, we calculated the standard deviation across trials of the signal of interest. This time course was normalized to its mean value in the 100 millisecond prestimulus period and expressed in percent change from this value. A decrease in TTV indicates a negative correlation between spontaneous and evoked activity, while an increase in TTV cannot distinguish between no correlation and positive correlation^25^.

### Time-frequency decomposition and definition of frequency bands

Time-frequency decomposition was carried out using the wavelet transform, as implemented in Fieldtrip. Three cycles of the default Morlet wavelet were used to estimate 50 logarithmically-spaced frequencies from 2 to 200 Hz (or from 2 to 50 Hz in the EEG case). The delta band was defined as 2–4 Hz, low gamma as 30–100 Hz (or 30–50 Hz in the case of the EEG dataset), and high gamma as 100-200 Hz. Since alpha peak frequency and peak width varies significantly between individuals, we followed the recommendations of Klimesch^97^ and defined the alpha, theta, and beta bands individually for each subject. We estimated the alpha peak frequency and width using the toolbox developed by^98^. The alpha band was then defined according to this width: the theta band was subsequently defined as 4 Hz to the lower bound of the alpha band, and beta was defined as the upper bound of the alpha band to 30 Hz. For subjects where no alpha peak was found, these bands were defined in the standard way (theta as 4–8 Hz, alpha as 8–13 Hz, and beta as 13–30 Hz).

### Separation of oscillatory and fractal components of evoked spectral power

To distinguish oscillatory and fractal processes in the spectral power response, we used the Irregular Resampling for Auto-Spectral Analysis (IRASA) method, described in^57^. In brief, this method involves resampling the signal using a variety of non-integer resampling factors and taking the median of the resulting power spectra to represent the fractal component. For this analysis, we used a subset of subjects from the main CamCAN dataset (*n* = 49), due to the extreme computational intensiveness of the time resolved IRASA procedure. Preprocessing for these subjects differed slightly from the above: due to the potential effect of filtering artefacts on the IRASA procedure, the IRASA data follows the preprocessing scheme described in the supplement (Fig. S12), where the initial 1 Hz highpass filter was omitted, and only the 0.03 Hz highpass filter implemented in the Elekta system at the time of acquisition was used.

Following a previous publication^85^, we used resampling factors ranging from 1.1 to 2.9 in steps of 0.05, excluding the factor 2. To assess stimulus-related changes in oscillatory and fractal power, we computed this method in a sliding window of size 1.5 s and step size 20 ms. This allowed us to estimate fractal and oscillatory power spectra across a range of 2 to 85 Hz. The oscillatory frequency bands for this process were the same as for the wavelet-based procedure, but since only frequencies up to 85 Hz could be resolved using the IRASA method, low gamma was defined as 30 to 50 Hz and high gamma as 50 to 85 Hz.

Scale-free activity can be defined as activity possessing a distribution of the form 1/f^β^ ^88,99^. As such, the fractal scaling exponent β was estimated as the slope of a linear least-squares fit of power versus frequency on a log-log scale; in the IRASA toolbox, this is computed by interpolating the data in order to be linearly spaced on the log-log scale, in order not to bias the estimation by the larger number of high-frequency estimates^57^. The broadband offset, which reflects the amplitude of scale-free fluctuations, was then defined as the y-intercept of this same linear fit. As the broadband offset is a power value, we exponentiated it prior to applying the method of pseudotrials. For the supplementary analyses in which the fractal power was analyzed in different frequency bands, this was calculated simply as the mean power within each frequency range, exactly like the oscillatory power. Note that despite this parametrization of the fractal power in different bands, the IRASA procedure was run on the raw data, as detailed above.

Occasionally, the IRASA method returns negative values of oscillatory power because oscillatory power is computed as the difference of the mixed power spectrum and the fractal component (which, due to its estimation as a median of resampled power spectra, can occasionally be larger in magnitude than the original mixed PSD). Negative values of oscillatory power were treated as missing data in this study.

### Statistics and reproducibility

In order to achieve good statistical power and flexibility without a strong *a-priori* hypothesis as to the latency or topography at which a correlation between spontaneous and evoked activity might manifest, we opted to used cluster-based permutation tests^100^. Our time window of interest was defined as the time between 0 and 800 milliseconds poststimulus. We tested for significant difference between the corrected prestim low and high time courses using a Wilcoxon signed-rank test at each time point and each sensor, using a cluster-based permutation test with 10,000 permutations to correct for multiple comparisons. Note that the resolution of the p-value is limited by the number of permutations: numbers of permutations from 1000^70,101^ to 10,000^102^ have been reported in the literature. We chose the upper number as many of our effects were highly significant, and frequently achieved the minimum possible p-value even with this higher number of permutations: however, to reduce computational burden, for our simulation analyses and analyses reported in the supplementary materials we performed only 1000 permutations.

The cluster procedure works by summing the test statistics of all significant tests in the time window which are adjacent in time and space (i.e. neighboring time points and sensors). To generate a *p*-value, this summed cluster statistic is then compared with a permutation distribution built from resampling the data a number of times and repeating the same procedure. For the TTV-based method, we used a signed-rank test against zero, similarly using a cluster-based permutation test to address the multiple comparison problem. We also calculated effect size (Cohen’s *d* for dependent samples, calculated as the mean difference divided by the standard deviation of the difference^103^) at each sensor and time point, and report the average of these effect size estimates over all points within each significant cluster.

Summary indices of the magnitude of spontaneous-evoked correlation were then calculated only for the sensors and time points which formed part of a significant cluster, in both the pseudotrial and TTV methods. However, we note that the permutation test does not establish the significance of the latency or topography, per se—we considered it here merely as a useful prior for calculating a summary index of the magnitude of spontaneous-evoked correlation. Summary indices were calculated at each sensor by taking the area under the curve of the difference between prestimulus high and low or the TTV time course across all time points in a significant cluster. These indices were then correlated across subjects at each sensor using Spearman’s rank correlation: significance of these correlations was likewise computed with a cluster-based permutation test, clustering across sensors. Permutations were carried out by randomly exchanging conditions in the signed-rank tests, and by randomly shuffling subjects for the correlation statistics.

As stated above, the sample size for the MEG dataset was 474, and the sample size for the EEG dataset was 26.

### Simulation of the discrepancy between pseudotrial method and TTV method

It has recently been observed that in EEG, TTV and spectral power are closely related^55^. This could present a confounding factor for the assessment of spontaneous-evoked correlation in the time-domain signal. To assess this possibility, we carried out two simulations: true correlation between spontaneous and evoked activity in the time-domain signal, and oscillatory power reduction with no additional time-domain stimulus response. Forty-eight subjects and 128 4-second long trials were simulated using an in-house modification of Fieldtrip’s ft_freqsimulation function. In each trial, electrophysiological data were simulated as a summation of 1/f^β^ noise (β randomly chosen between 0.5 and 1.5) and an alpha oscillation at 10 Hz. We modeled the amplitude of this oscillation itself as a 1/f^ß^ process lowpass filtered at 1 Hz. In the spontaneous-evoked correlation simulation, a stimulus related increase in the form of one lobe of a sine function was added to the signal; the magnitude of this increase was varied according to the value of the prestimulus voltage. In the oscillatory power reduction simulation, the amplitude of the 10 Hz oscillation was reduced over the same time period, again with the response taking the form of one lobe of a sine function. We then tested for a correlation between spontaneous and evoked activity using the method of pseudotrials and the method of TTV, as described above. As in our real data, we used a cluster-based permutation test, clustering only across the time dimension as only one channel was simulated.

### Simulation of additive and non-additive models accounting for trial-varying signal-to-noise ratio

A correlation between spontaneous and evoked activity could conceivably be observed due to trial-varying signal-to-noise ratio. If SNR is high on some trials, one may observe an association between higher prestimulus power and a greater evoked response simply due to the evoked power estimation being less corrupted by noise in these trials. To test for this possibility, we conducted simulation experiments in which we systematically varied the signal-to-noise ratio across trials (Figs. S2 and S3). We varied both the base SNR and the across-trial variability of the noise across four orders of magnitude (from 1/64 to 64 in each case). Under each noise regime, we tested two models: one, in which the evoked response and the ongoing dynamics were independent (negative control; Fig. S2), and another in which the evoked response and ongoing dynamics were correlated (positive control; Fig. S3). The simulation was carried out as described above for the oscillatory power reduction simulation (though the evoked response was extended to better match the observed temporal characteristics of the alpha response). The magnitude of this decrease was either scaled by the spontaneous amplitude in the 100 millisecond prestimulus period (positive control; spontaneous-evoked correlation) or varied randomly (negative control; additive relationship between spontaneous and evoked activity). Statistical tests were carried out as described above.

### Reporting summary

Further information on research design is available in the Nature Research Reporting Summary linked to this article.

## Supplementary information

Supplementary Information

Reporting Summary

## Data Availability

MEG data are available from the CamCAN repository (https://camcan-archive.mrc-cbu.cam.ac.uk/dataaccess/). Data from the EEG replication study are available from the corresponding author on reasonable request. Source data underlying the figures is available at 10.5281/zenodo.4724018.

## Code availability

Custom scripts were used for the analysis of correlation between spontaneous and evoked activity; these are available from GitHub (https://github.com/SorenWT/spontevo2020) and Zenodo (10.5281/zenodo.4747781)^104^.
