# Supplementary material for: Dynamic relationships between spontaneous and evoked electrophysiological activity
Source: Commun Biol. 2021 Jun 15;4:741. doi: 10.1038/s42003-021-02240-9 (PMC8206204; doi:10.1038/s42003-021-02240-9)
Supplement: Supplementary file 1 — Supplementary Information [file 42003_2021_2240_MOESM1_ESM.pdf]

Supplementary materials

Spectral power response of fractal and oscillatory components

While the IRASA method has been applied in the context of continuous, long-term EEG and MEG recordings[1,2], it has not been applied in a time-resolved manner to stimulus related activity. Here we report (in brief) the stimulus-related changes of oscillatory power and fractal power in each frequency band; these results will be reported in more detail in a forthcoming publication (Wainio-Theberge et al., in preparation).

We observed a significant spectral power change in each frequency band, in both the fractal and oscillatory components ($p \leq 0.002$ in each band and fractal parameter). The spectral power response of the oscillatory component was familiar; it shows an increase in delta- and theta-band activity which peaks early in the post-stimulus period (250 ms), and a decrease in theta-, alpha-, beta-, and low gamma-band activity which bottoms out later in the post-stimulus period (400-600 ms). High gamma band activity showed a very small post-stimulus increase. In contrast, both of the fractal parameters (slope and intercept) increased following stimulus onset. These results and their implications will be discussed in greater detail in a forthcoming publication.

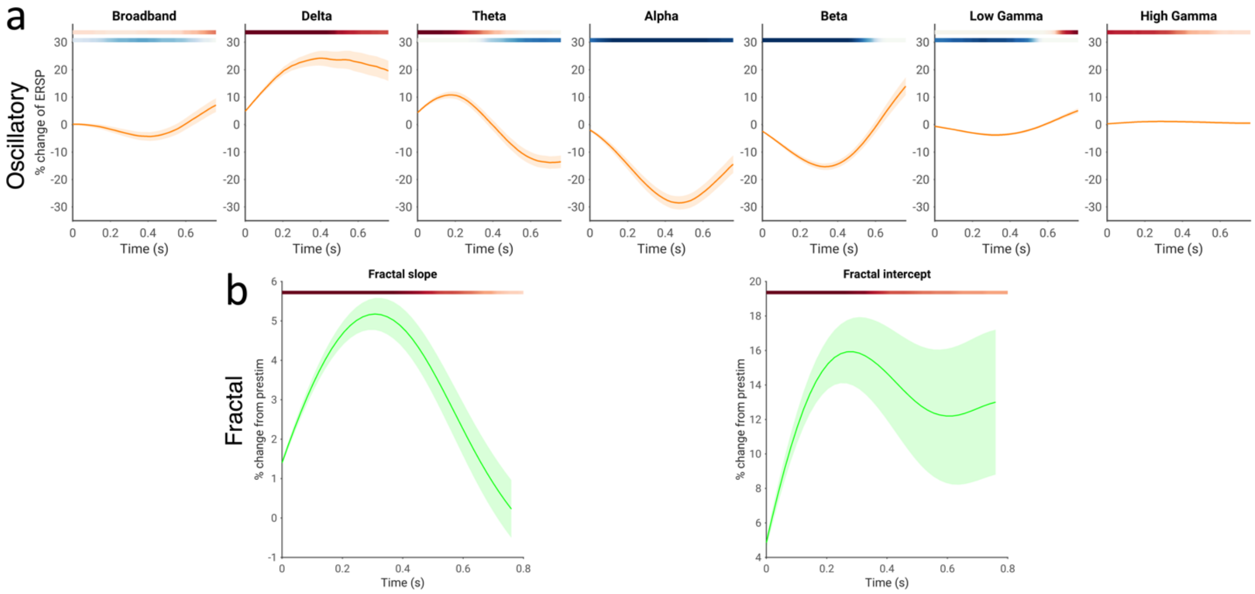

Figure S1. Spectral power response of oscillatory power and fractal parameters. Asterisks indicate a significant difference from zero. Shading indicates standard error. * = $p < 0.05$, ** = $p < 0.01$.

Validation of the pseudotrial and TTV methods in simulated negative-correlation and no-correlation scenarios – the effect of trial-varying signal-to-noise ratio

It is possible that trial-by-trial variations in signal-to-noise ratio (SNR) could generate spurious evidence of a correlation between spontaneous and evoked activity. This could occur as trials with high SNR will show a large evoked response, while trials with low SNR may show a

smaller evoked response. We tested for this possibility using simulations, which are described in more detail in the methods.

Figure S2 shows the result of the additive simulation, in which no correlation between spontaneous and evoked activity was present. Only one SNR regime showed a significant correlation using the pseudotrial method. For 49 SNR conditions, the expected number of false positives is 2.45, so this is well within the expectation under chance conditions ($p = 0.7101$, binomial test). Furthermore, these effects were small, confined to short periods of time, and do not qualitatively resemble at all the robust effects observed in our data. Using the TTV method, eight SNR regimes showed significant decreases of TTV. This is significantly more than should be expected by chance alone ($p = 0.000648$, binomial test). It appears that the TTV method may be adversely affected by variations in signal-to-noise ratio, particularly in certain noise regimes; however, we note that the effects observed in this data are rather small in comparison to the TTV reductions observed in the empirical data. Several other conditions showed a significant increase in TTV in the post-stimulus period – we do not consider these results in the statistics, since, as discussed in the methods, an increase in TTV is to be expected in purely additive conditions and is not indicative of a correlation between spontaneous and evoked activity.

Figure S3 shows the result of the simulation in which a negative correlation between spontaneous and evoked activity was introduced. Using the method of pseudotrials, 17 SNR conditions showed significant effects in the expected direction ($p = 9.82 \times 10^{-12}$, binomial test). A further two displayed limited evidence of a positive correlation, rather than a negative one ($p = 0.2139$, binomial test with $n = 32$). These conditions were generally the high SNR and low SNR variability conditions, as should be expected. Using the method of TTV, 28 conditions showed significant TTV decreases ($p = 0$, binomial test). Again, these conditions usually had high SNR and low SNR variability, but TTV decreases were visible in lower SNR conditions than for the method of pseudotrials.

Taken together, these results suggest that the method of pseudotrials is a robust and accurate method for assessing spontaneous-evoked correlation and is not subject to false positives due to time-varying noise. The method of TTV, by contrast, appears to have greater power to detect negative correlation than the pseudotrial method, but it occasionally exhibits false positives due to trial-by-trial variations in noise contamination.

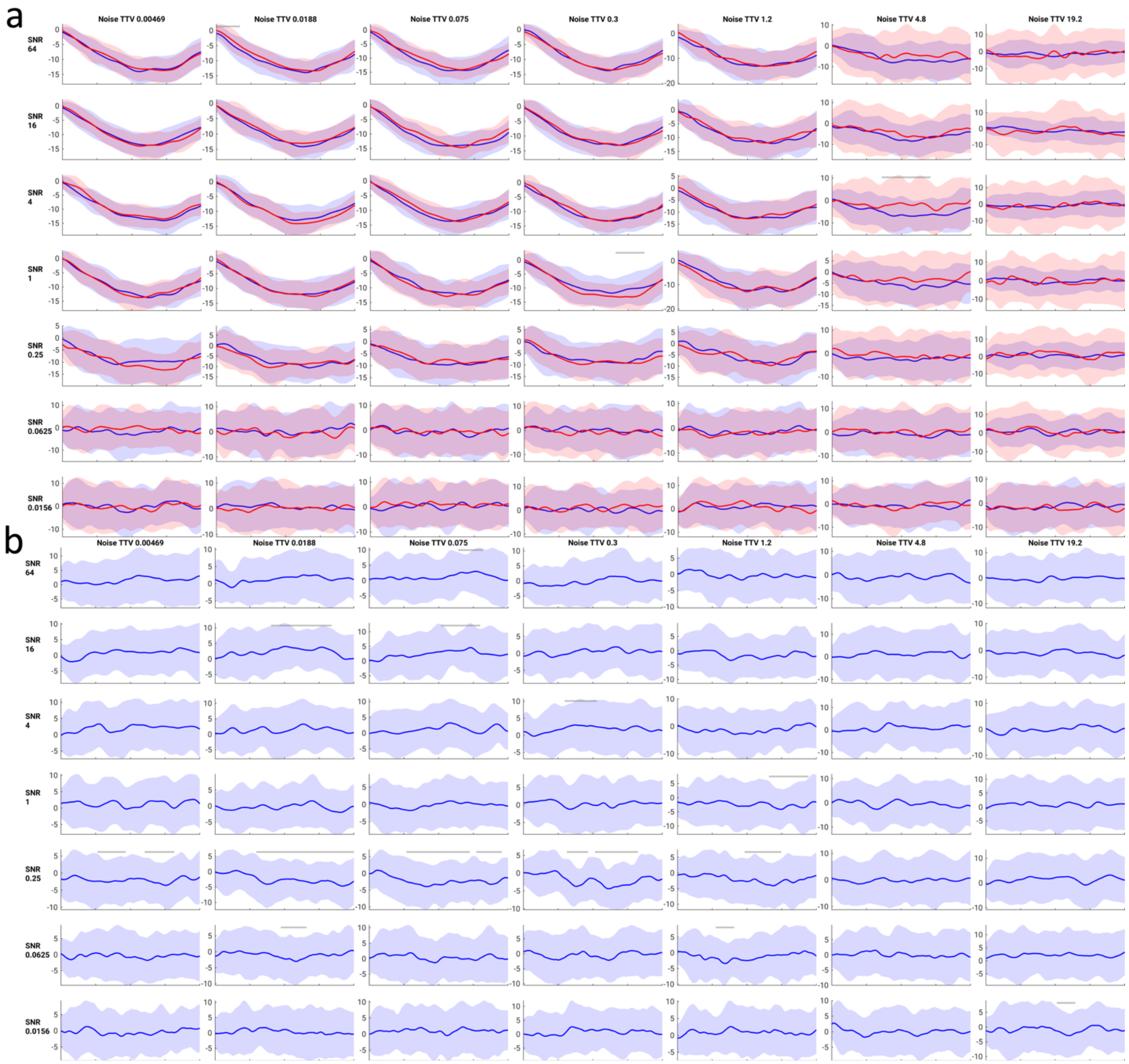

Figure S2. Results of the negative control simulation study, using both the method of pseudotrials and the method of trial-to-trial variability. **a**) Pseudotrial-based methodology. Rows indicate different average signal-to-noise ratios, and columns indicate different variabilities of noise across trials. Each plot shows the pseudotrial-corrected time course for the high prestimulus (red) and low prestimulus (blue) conditions. Shaded overbars indicate significance following a cluster-based permutation test (clustering across time only). **b**) TTV-based methodology. Each plot shows the TTV time course – shaded overbars indicate significance as in (**a**).

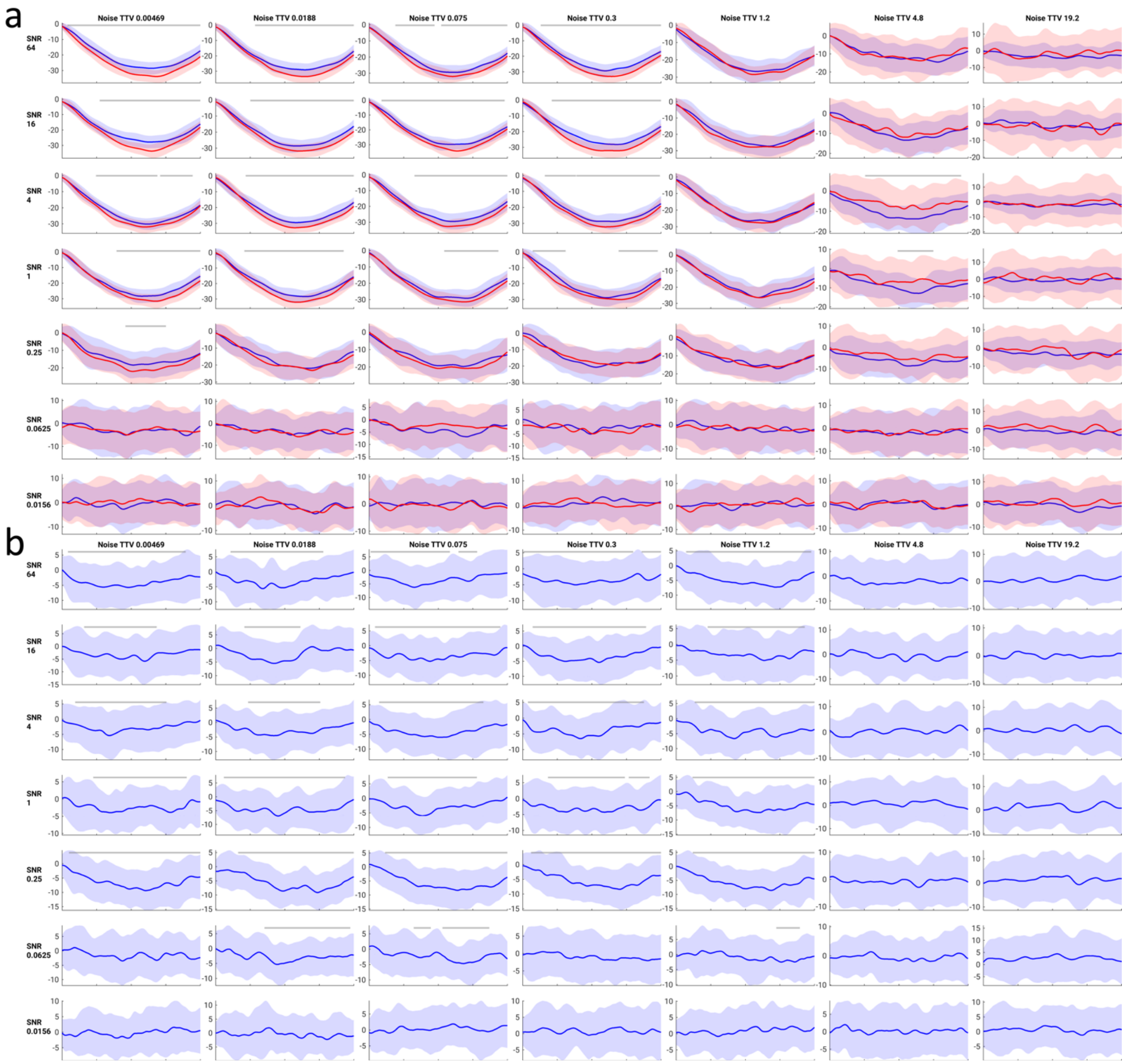

Figure S3. Results of the positive control simulation study, using both the method of pseudotrials and the method of trial-to-trial variability. **a**) Pseudotrial-based methodology. Rows indicate different average signal-to-noise ratios, and columns indicate different variabilities of noise across trials. Each plot shows the pseudotrial-corrected time course for the high prestimulus (red) and low prestimulus (blue) conditions. Shaded overbars indicate significance following a cluster-based permutation test (clustering across time only). **b**) TTV-based methodology. Each plot shows the TTV time course – shaded overbars indicate significance as in (**a**).

Assessing the effect of prestimulus length – 50 and 200 millisecond prestimulus intervals

We performed control analyses to ensure that the length of the prestimulus window chosen did not substantially affect the results of our analyses. Different frequency bands have different cycle durations, and as such it is possible that different lengths of the prestimulus period

may affect the accuracy by which one can assess power within that window. We tried a 50 millisecond and a 200 millisecond prestimulus window, in addition to the 100 millisecond window in the main text, and replicated the analyses of figures 2 and 3 from the main text. The results were almost entirely consistent with the results of the main text.

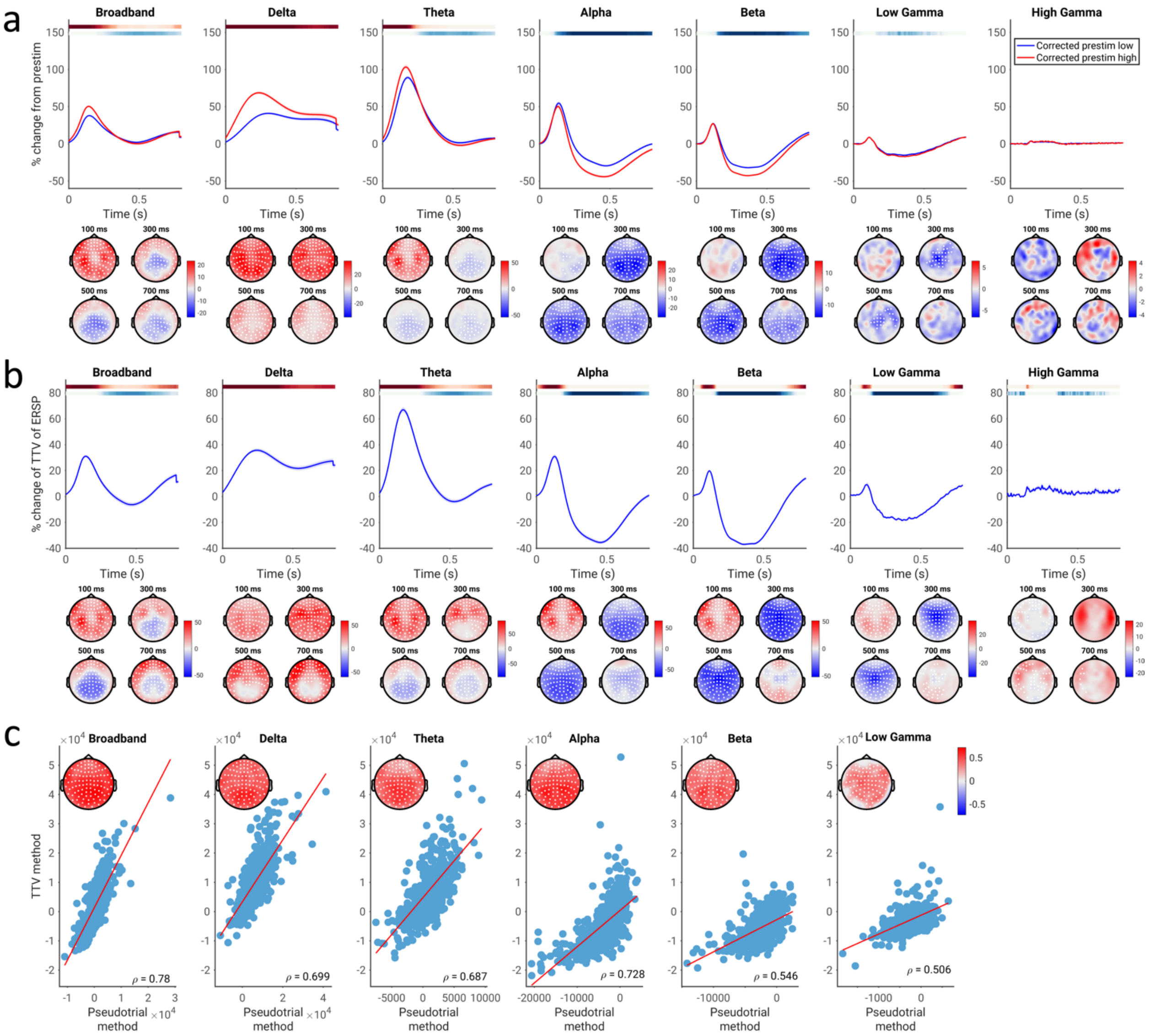

Figure S4. As figure 2 in the main text but using a 50 millisecond prestimulus window.

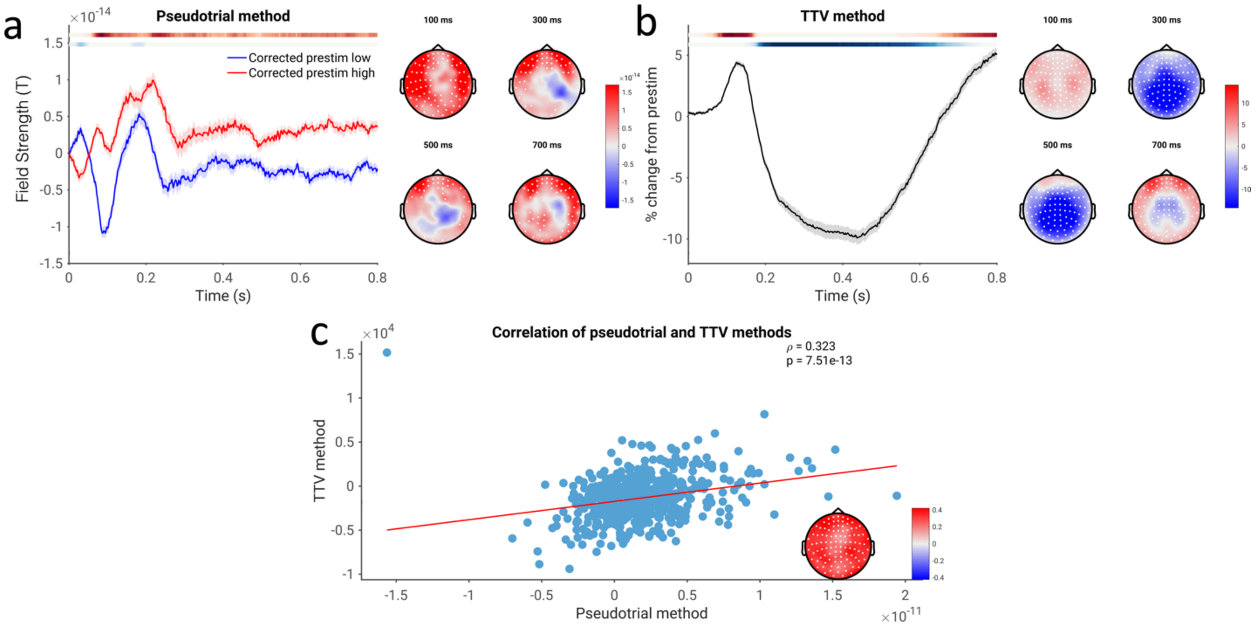

Figure S5. As figure 3 in the main text, but using a 50 millisecond prestimulus window.

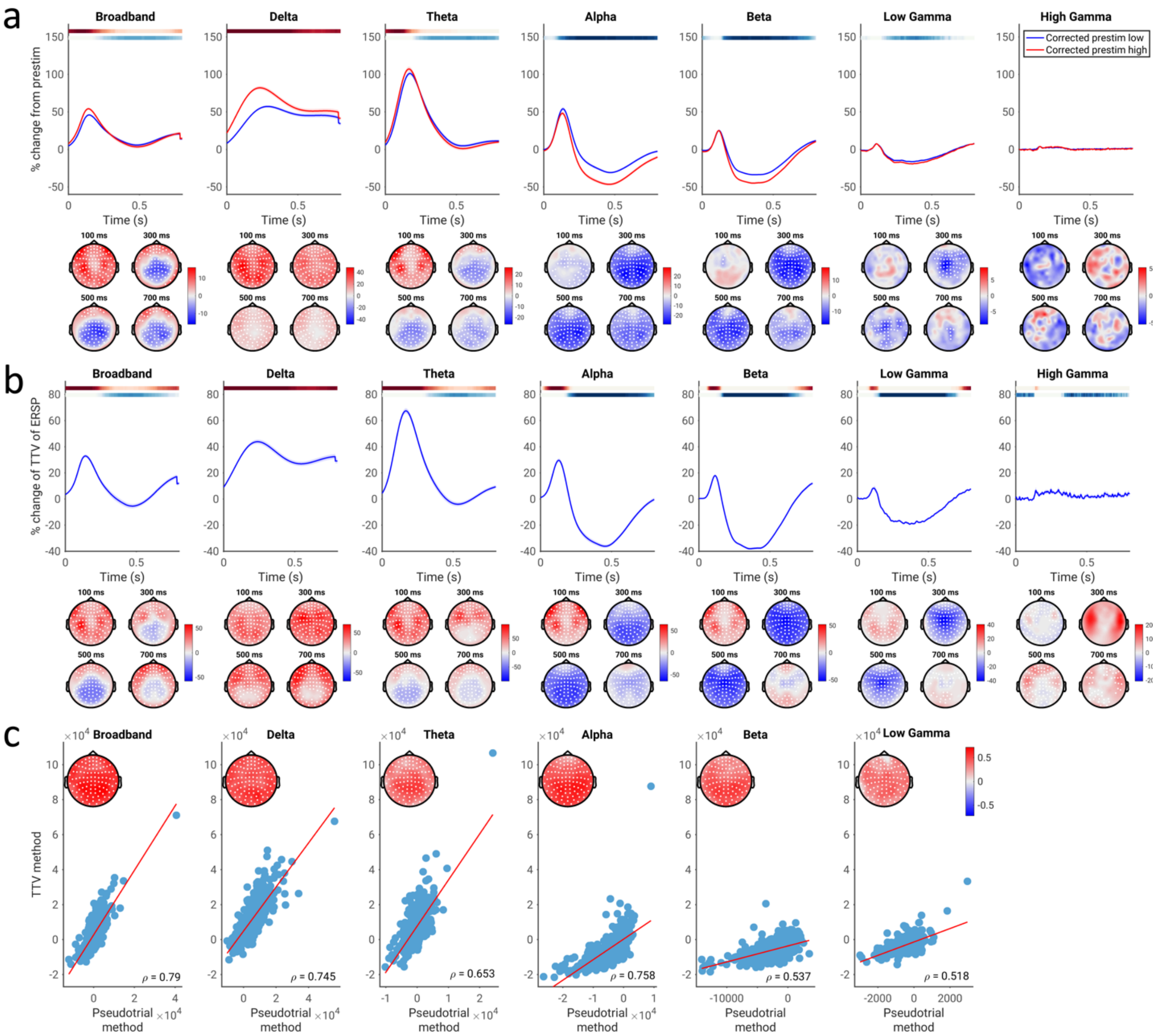

Figure S6. As figure 2 in the main text but using a 200 millisecond prestimulus window.

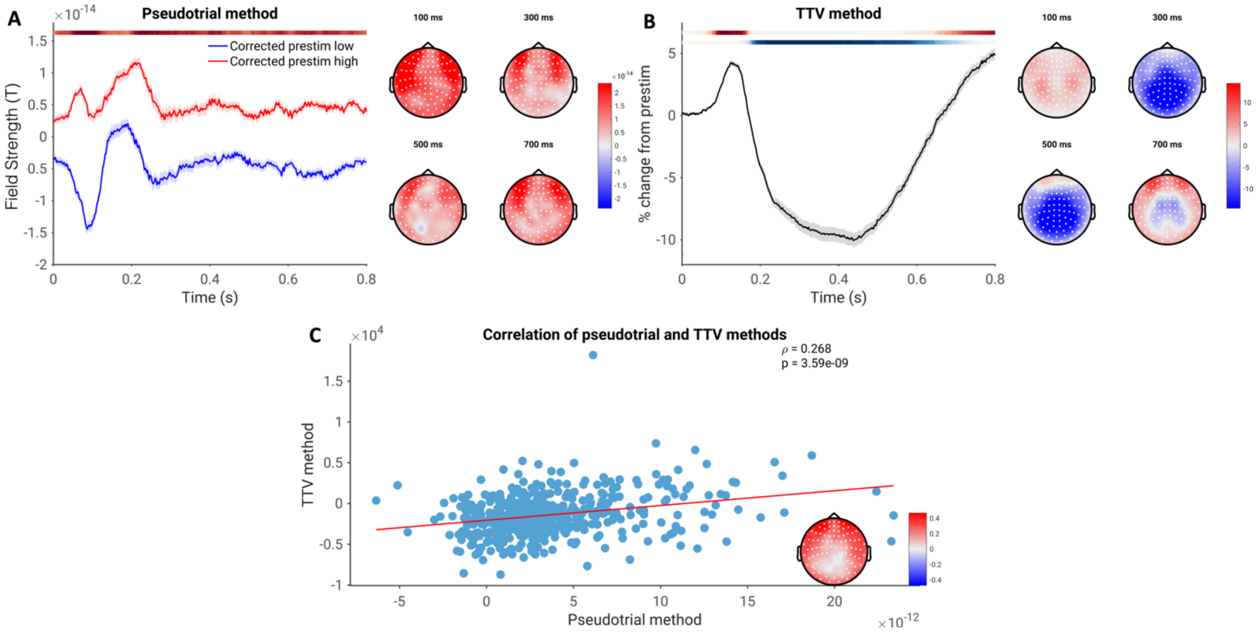

Figure S7. As figure 3 in the main text but using a 200 millisecond prestimulus window.

Controlling for the influence of anticipation

We chose to place the pseudotrials in directly antecedent to the real-qtrial prestimulus period in order allow for epoch correction using AutoReject, and to make use of as many trials as possible in the CamCAN dataset (though the ITI in this dataset ranges from 2 to 26 seconds, the majority of trials have ITIs less than 5 seconds). However, this choice may result in anticipation effects, whereby the pseudotrial does not reflect genuinely "spontaneous" activity. We controlled for this by repeating the analysis with the following procedure. First, we selected trials with ITIs of 5 seconds or longer (median number of trials per subject = 32, SD = 1.57). The pseudotrial period (lasting 900 ms) was then drawn at random from the period between 2 seconds after the preceeding stimulus and 2 seconds before the present stimulus. We then carried out the same analyses as in the main text for frequency- and time-domain data.

In the frequency domain, results were generally similar to the results using the original pseudotrial procedure. However, in the time-domain data, the positive correlation assessed with the method of pseudotrials was much reduced (though still statistically significant: $p \leq 0.0120$, average $d = 0.127$). This result further underscores the relative insensitivity of spontaneous-evoked correlation in the frequency domain to analytical parameters, and the sensitivity of time-domain spontaneous-evoked correlation to variations in task, modality, or analytical procedure. It may also be that the time-domain spontaneous-evoked correlation is simply much noisier than the frequency-domain correlations, and 32 trials per subject is not sufficient to resolve it. Alternatively, it may be that anticipation effects are at play in either the correlation of spontaneous and evoked activity, or the response itself, as ERP components have been shown to be modulated by expectancies[3,4].

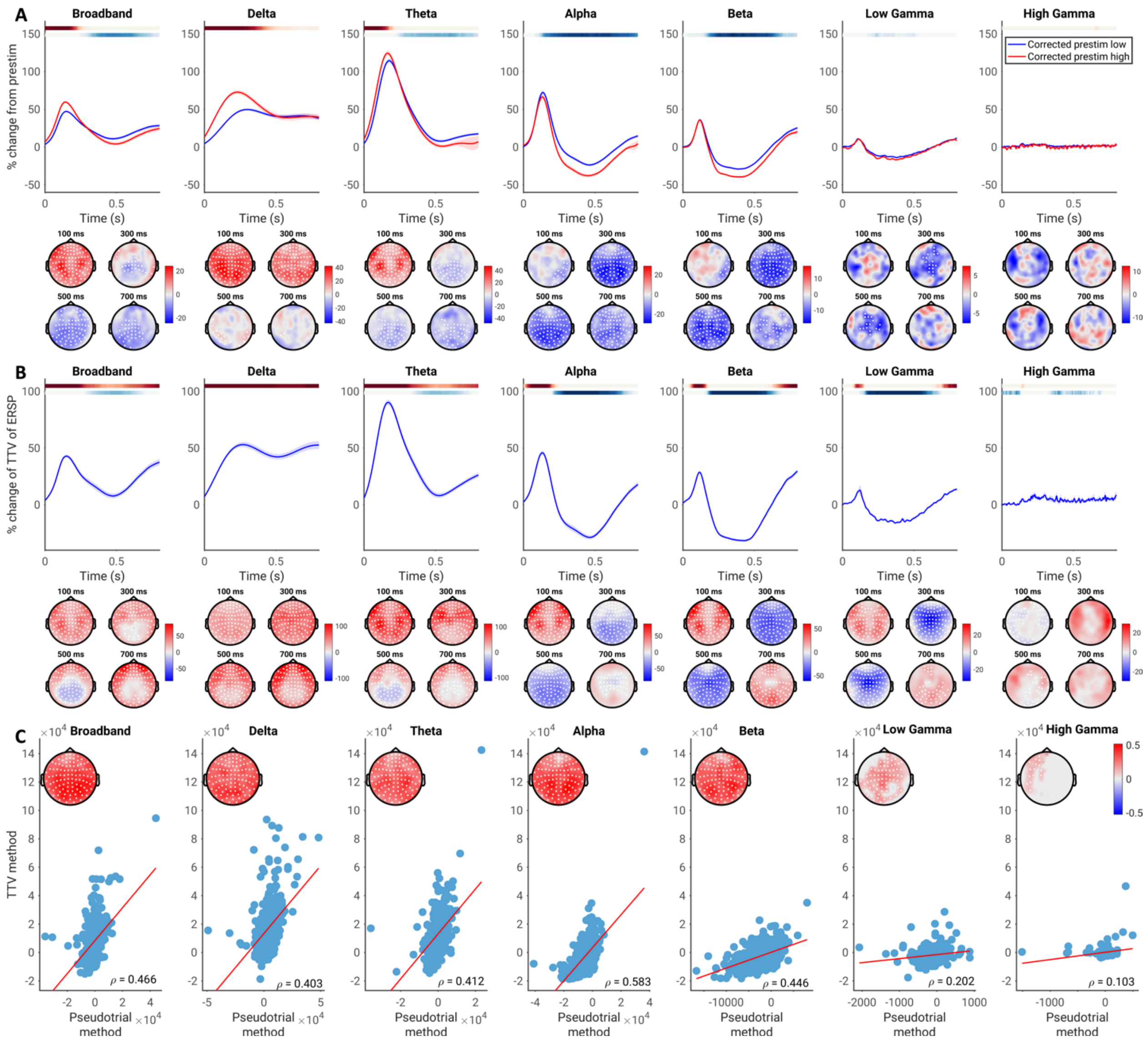

Figure S8: As figure 2 in the main text, but with pseudotrials drawn randomly from a period not directly antecedent to the real-trial prestimulus period.

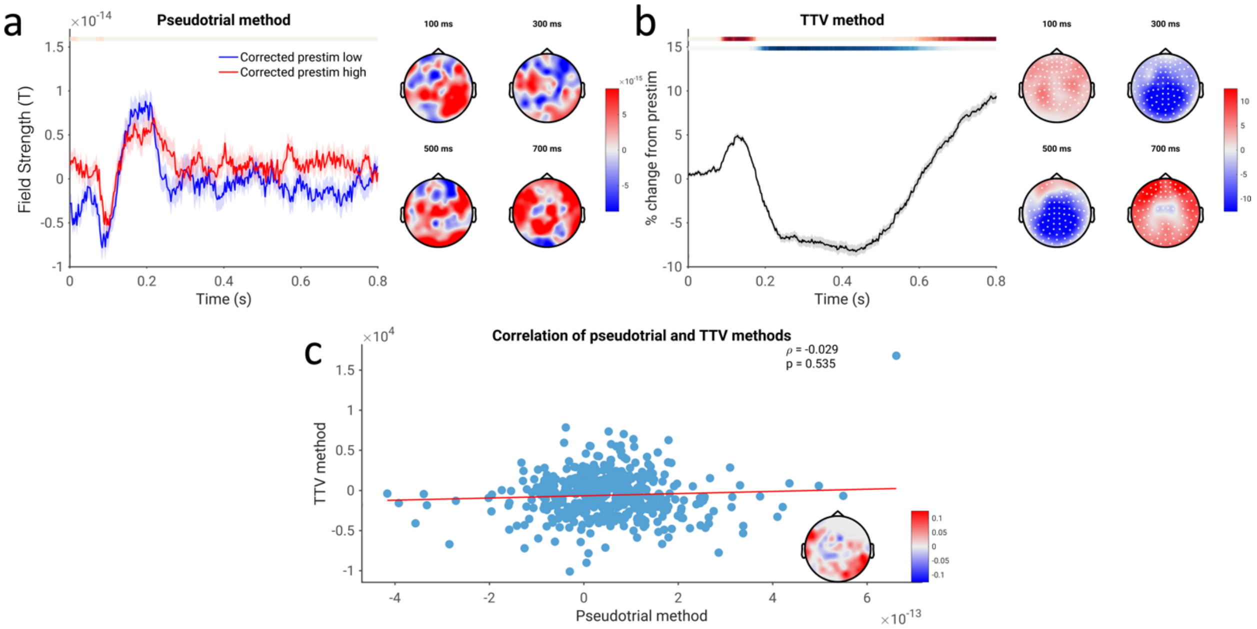

Figure S9: As figure 3 in the main text, but with pseudotrials drawn randomly from a period not directly antecedent to the real-trial prestimulus period.

Spontaneous-evoked correlation of oscillatory and fractal dynamics using the method of TTV

In the main text, spontaneous-evoked correlation of oscillatory and fractal dynamics was assessed using the method of pseudotrials. Here we show that broadly analogous results hold using the method of TTV. For oscillatory power, we observed a TTV increase in delta ($p \leq 0.002$, average $d = 0.674$) and theta ($p \leq 0.002$, average $d = 0.652$), consistent with the positive correlation observed with the method of pseudotrials. The subsequent decrease in TTV in theta was not significant ($p = 0.745$, average $d = 0.639$). In alpha, beta, and low gamma, we observed a TTV decrease ($p \leq 0.002$ in each case; alpha: average $d = 0.996$; beta: average $d = 1.07$; low gamma: average $d = 0.590$), consistent with the negative correlation observed using the pseudotrial method. Interestingly, the fractal slope and intercept displayed both TTV increases ($p \leq 0.0002$ in each case; average $d = 0.498$ for slope, 0.549 for intercept) and TTV decreases over central sensors partway through the poststimulus period ($p = 0.0154$ for slope, $p = 0.0132$ for intercept; average $d = 0.429$ for slope, 0.381 for intercept). In the case of broadband fractal power, the pseudotrial method finds similar results, though they do not reach significance, in keeping with the greater sensitivity (but reduced specificity) of the TTV method. However, the methods are in disagreement for the fractal slope, where significant positive correlation was found with the method of pseudotrials for the same sensors and time points.

For most major effects, the TTV method and the pseudotrial method were in agreement for the oscillatory and fractal component data; however, there are a few notable differences. We submit that these differences may be due to overestimation of positive correlation using the method of pseudotrials (see figure S10 for details).

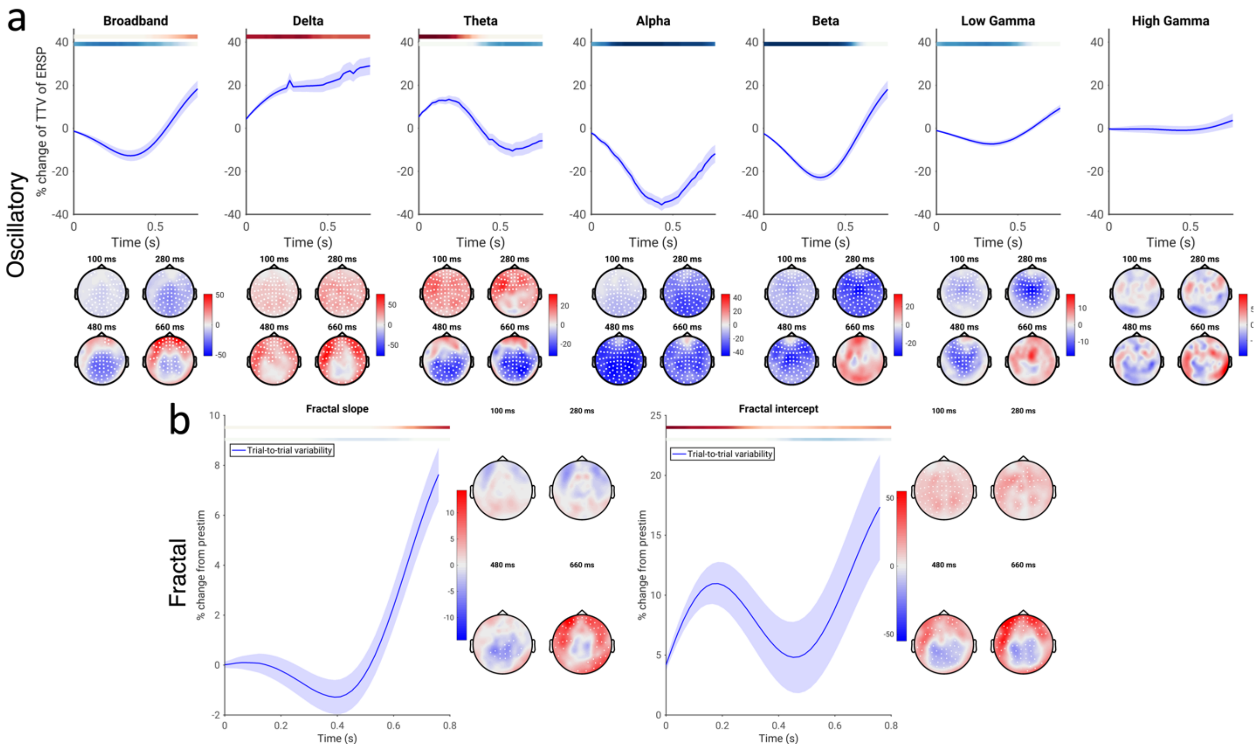

Figure S10: Spontaneous-evoked correlation of oscillatory (**a**) and fractal (**b**) power, assessed using the method of trial-to-trial variability. Shaded area indicates standard error. Effect topographies are shown at 100 ms, 300 ms, 500 ms, and 700 ms. * = $p < 0.05$, ** = $p < 0.01$.

Spontaneous-evoked correlation of fractal power in oscillatory bands

While scale-free activity is best understood in terms of the scaling exponent and broadband offset described in the main text, such an analysis makes it difficult to directly compare scale-free and oscillatory activity. In order to determine which of these parameters exhibited stronger correlations between spontaneous and evoked activity, and thus to shed light on the differential impacts of scale-free activity and cortical oscillations on the mixed power spectrum, we undertook a somewhat unconventional analysis of scale-free activity by analyzing it in terms of the mean power within each of the oscillatory frequency bands. While this may seem counter to the interpretation of the fractal component as a scale-free process which is not confined to a particular narrow frequency band, it can be considered as an alternate parametrization, akin to binning a continuous variable. Fractal power in different frequency bands is calculated as the mean of fractal power within that band – this results in a parametrization which, while less parsimonious than the slope-intercept view, allows for direct comparison between fractal and oscillatory power (Figure S11A - see [5] for a similar approach).

Similar to the oscillatory and mixed power, the fractal component in the delta and theta bands displayed a positive correlation between spontaneous and evoked values (Figure S11B; $p \leq 0.0002$, average $d = 0.670$ and 0.554, respectively), and beta and low gamma displayed a negative correlation ($p \leq 0.0002$, average $d = 0.549$ and 0.502, respectively). However, it is noteworthy that in the fractal component, the alpha band displayed a small positive correlation, rather than a negative one as observed in the oscillatory component ($p = 0.001$, average $d = 0.444$). Once again, high gamma displayed a significant but small positive correlation ($p = 0.001$, average $d = 0.348$). Finally, we attempted to directly compare the magnitudes of spontaneous-evoked correlation between the fractal and oscillatory components. We find that in most cases the oscillatory component displayed a much stronger correlation between spontaneous and evoked activity, particularly in the delta, theta, alpha, and beta bands, as well as high gamma (Figure S11C; $p \leq 0.0002$ for all; delta: average $d = 0.556$; theta: average $d = 0.531$; alpha: average $d = 0.832$; beta: average $d = 0.578$; high gamma: average $d = 0.429$). The fractal component displayed a slightly stronger correlation in the low gamma band, but the effects were again small compared with the other bands ($p \leq 0.0002$; average $d = 0.468$). These results suggest that when compared head-to-head, cortical oscillations show stronger relationships between spontaneous and evoked activity than scale-free dynamics.

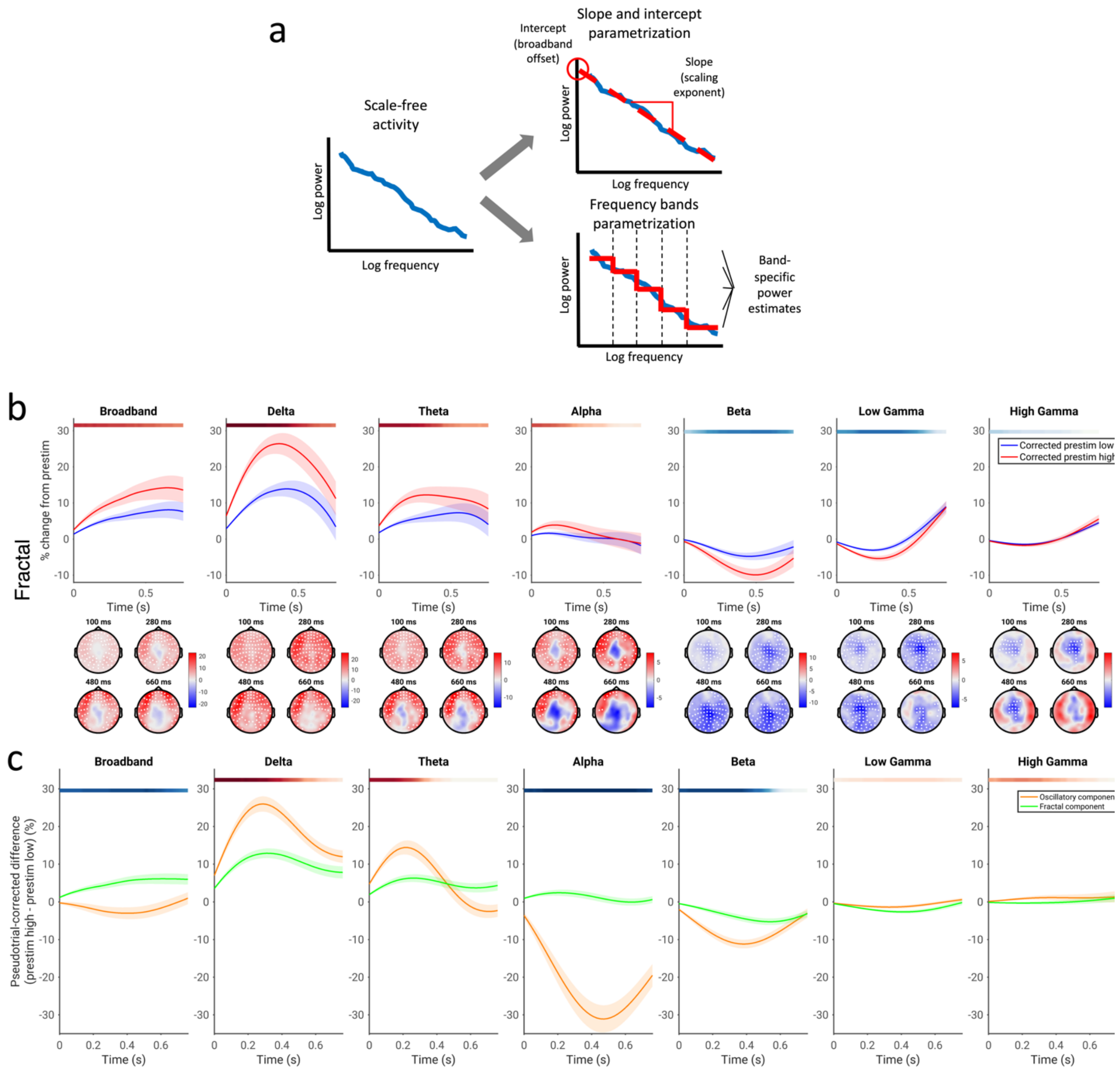

Figure S11. Direct comparison of spontaneous-evoked correlation of oscillatory and fractal activity. **a**) Schematic of the different ways of parametrizing scale-free activity used in the study. Scale-free activity is estimated using the IRASA method prior to either parametrization approach. Subsequently, it can be analyzed either in terms of slope and intercept (from a linear fit of power versus frequency on a log-log scale (top), or in terms of the average power in different frequency ranges (bottom). **b**) As figure 5a in the main text, but for fractal power subdivided into frequency bands. **c**) Magnitude of the spontaneous-evoked relationship over time for oscillatory and fractal components. Overbars as in **b**) indicate the significance of the difference between these spontaneous-evoked correlation estimates of these two components.

The effect of filtering on time-domain spontaneous-evoked correlation

It is known that high-pass filtering at 1 Hz can distort event-related potential (ERP) waveforms, altering their latency and amplitude[6]. In the main text, we chose to use highpass filtering at 1 Hz because this filtering best facilitates ICA decomposition, reducing the level of artefact contamination in the main results[7]. We report the analyses of spontaneous-evoked correlation on the unfiltered data here, in order to assess whether highpass filter distortion had an effect on the estimated correlation between spontaneous and evoked activity.

Preprocessing of this dataset was the same as described in the main text, but a copy of the data without the highpass filter was kept. The initial run of Autoreject, ICA training, and component classification were carried out on 1 Hz highpass-filtered data, but the weights from the ICA decomposition were applied back to the original, unfiltered data. Components classified as artefactual from this filtered data were then rejected in the unfiltered data. The remainder of preprocessing proceeded as described in the main text. n for this sample was slightly lower (n = 440 vs. 474) due to preprocessing errors.

Using the method of pseudotrials, we again noted a positive correlation between spontaneous and evoked activity in the time-domain signal ($p \leq 0.002$, average $d = 0.164$). However, in contrast to the main text results, this positive correlation was more limited in scope and magnitude, lasting only the first 200 milliseconds of the poststimulus period, rather than extending over the whole poststimulus period. Over central sensors, a negative correlation between prestimulus and poststimulus was observed between 300 and 400 milliseconds post-stimulus ($p \leq 0.002$, average $d = 0.148$). Results using the method of TTV were largely similar to the main text results: we observed a widespread decrease in TTV in the poststimulus period, as found in previous papers[8,9] ($p \leq 0.002$, average $d = 0.578$) as well as a brief TTV increase at approximately 100 ms poststimulus ($p = 0.0120$, average $d = 0.315$). The results of the two methods remained significantly correlated ($p \leq 0.002$), though this correlation remained small in magnitude.

These results suggest that 1 Hz highpass filtering may have exaggerated the magnitude of positive correlation in our data, and masked the presence of a topographically-limited negative correlation. This may have occurred if part of the ERP response was smeared backwards in time by the effect of filtering, as observed by[6]. This would have pushed part of the post-stimulus response into the pre-stimulus period, exaggerating the correlation between pre-stimulus and post-stimulus. We emphasize, however, that the finding of positive correlation remains significant even when this effect has been controlled for.

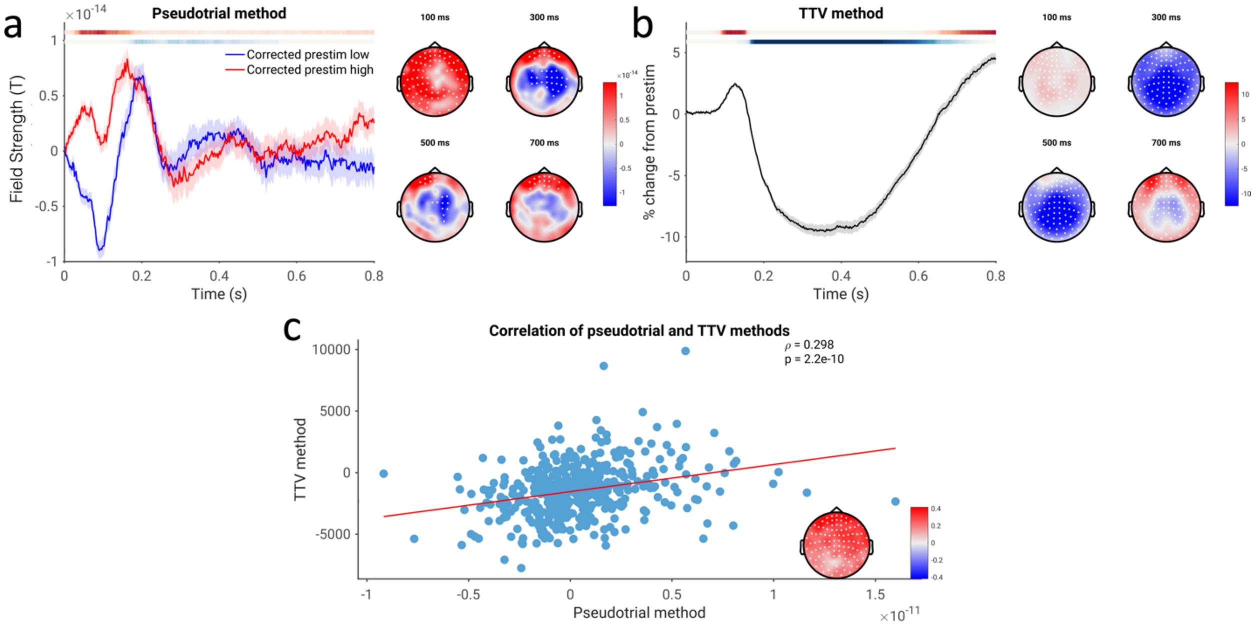

Figure S12. As figure 3 in the main text, but without the application of a 1 Hz highpass filter.

The effect of temporal resolution on spontaneous-evoked correlation in low frequencies

As discussed above in figure S9, any mechanism which results in part of the post-stimulus response being included in the pre-stimulus period may artificially inflate the magnitude of positive correlation observed. The estimation of low-frequency activity is inherently temporally imprecise, as the window size necessary to resolve low-frequency activity is large. As a result, it is possible that estimates of low-frequency activity in the prestimulus period are influenced by the poststimulus response.

We controlled for this with the following prodedure. First, we visualized the time course of the power of the lowest-frequency band included in our data (the delta band, 2-4 Hz), and determined the point at which it begins to rise (figure A). We then chose our prestimulus period as the 100 milliseconds before this observable rise: conservatively, this was -400 ms to -300 ms. We then set the pseudotrial prestimulus period at the same offset from the pseudotrial. As a result, we were only able to analyze a 400 millisecond post-stimulus period: however, since low-frequency activity was of interest, this should be sufficient, as the low-frequency responses we observed occurred early in the post-stimulus period.

We observed that the magnitude of positive spontaneous-evoked correlation in the delta and theta bands was substantially reduced, from a maximum channel-average difference of 33% in delta to a maximum channel-average difference of 6%. However, the effect remained highly significant both in the delta ($p \leq 0.002$, average $d = 0.155$) and theta bands ($p \leq 0.002$, average $d = 0.146$). Negative correlation estimates were largely unaffected. These results suggest that the magnitude of positive spontaneous-evoked correlation in slow-frequency activity may have been overestimated due to the temporal imprecision inherent in resolving this activity. Once again, however, we note that positive correlation remained significant despite controlling for this effect.

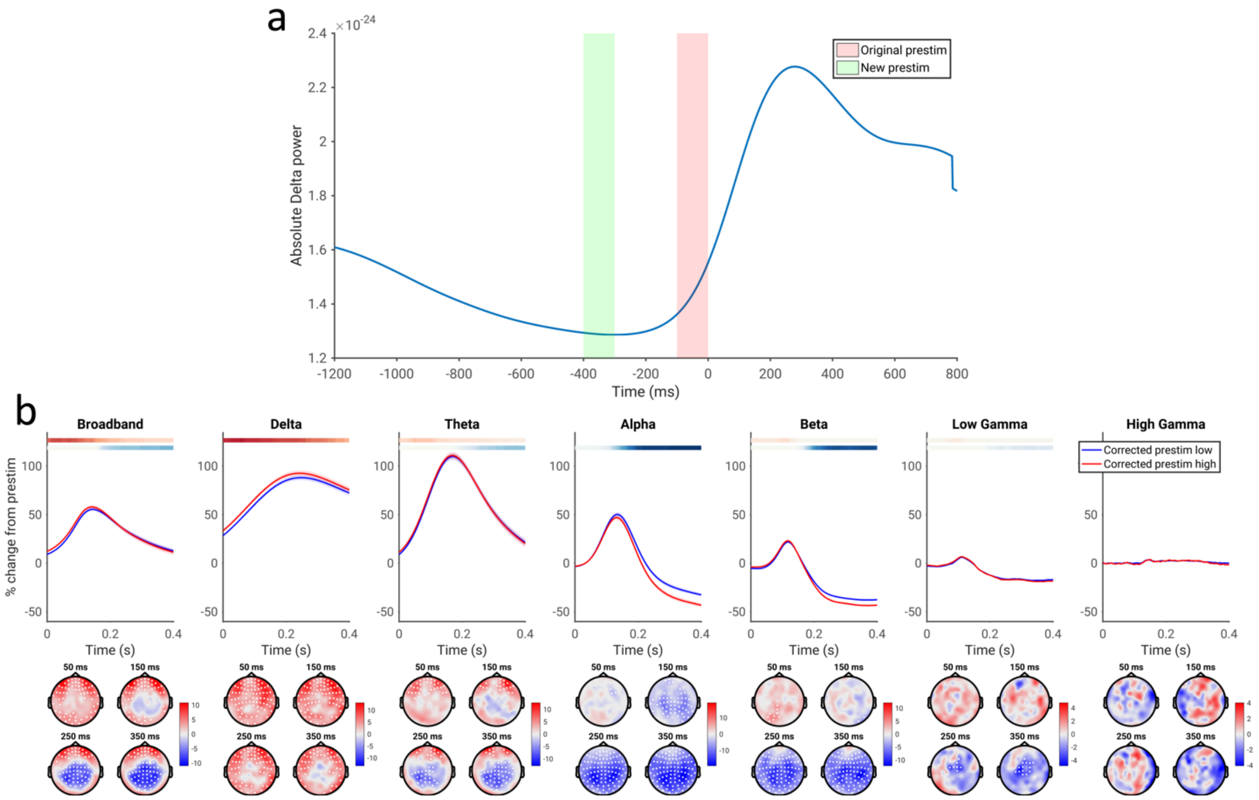

Figure S13: The effect of temporal resolution on low-frequency spontaneous-evoked correlation. **a**) The two prestimulus windows considered. The red highlight shows the original prestimulus window from the main text, while the green highlight shows the prestimulus window considered for panel **b**: note that this window falls before any increase in power is observed. **b**) Pseudotrial-corrected time courses and topoplots for analysis using the green prestimulus window from **a**. Asterisks, topoplots, and shading as in figure 2a) in the main text.

Time-domain correlation of spontaneous and evoked activity in different frequency bands

While the pseudotrial method and TTV method agreed well for estimating correlations between spontaneous and evoked activity in frequency-domain data, they found different results when applied to time-domain data. In the main text, we advanced the argument that TTV reductions in time-domain data can reflect changes in oscillatory power absent of genuine correlation between spontaneous and evoked activity. However, another possibility is that the method of pseudotrials is at fault. For time domain signals, where fluctuations are present at different scales, the prestimulus period of the method of pseudotrials may not be appropriate to capture up-and-down states for all bands: for example, 100 ms may be too long to capture a meaningful "prestimulus" period of high-frequency changes. While time-domain changes are traditionally analyzed in a broadband manner by identifying different ERP components, there is a growing understanding that different ERP components may reflect processes which are band-specific (ex. the P300 ERP may reflect event-related delta phase synchronization[10]). Thus, we analyzed the correlation of spontaneous and evoked activity in the time domain electrophysiological signal by dividing it into different frequency bands.

To do this, we used a filter bank approach: for each frequency band defined in the main text, we filtered the MEG data using a 4th-order Butterworth filter. We then computed indices of spontaneous-evoked correlation using both methods, as described in the main text.

Considering the filtered ERP data, we observed significant positive correlations in delta, theta, beta, and low gamma using the method of pseudotrials ($p \leq 0.002$ for each; delta: average $d = 0.295$; theta: average $d = 0.263$; beta: average $d = 0.166$; low gamma: average $d = 0.119$). We also observed significant negative correlations in delta, theta, and alpha ($p \leq 0.002$ for each; delta: average $d = 0.195$; theta: average $d = 0.159$; alpha: average $d = 0.172$) – these negative correlations were interleaved with the positive ones in delta and theta, suggesting phase-related effects. Using the TTV method, we observed TTV increases in delta, theta, beta, low gamma and high gamma ($p = 0.004$ for low gamma, $p \leq 0.002$ otherwise; delta: average $d = 0.544$; theta: average $d = 0.569$; beta: average $d = 0.377$; low gamma: average $d = 0.171$; high gamma: average $d = 0.182$), as well as TTV decreases in theta, alpha, beta, and low gamma ($p \leq 0.002$ for each; theta: average $d = 0.343$; alpha: average $d = 0.952$; beta: average $d = 1.17$; low gamma: average $d = 0.224$). Once again, the metrics were weakly correlated in all bands, though alpha and delta achieved moderate correlations (channel-average $\rho = 0.571$ and $\rho = 0.424$). While the filtering procedure resulted in agreement between the TTV and pseudotrial methods in alpha (and non-contradiction in delta and theta), discrepancies remained in the beta and low gamma bands, where the pseudotrial method indicated a positive correlation between spontaneous and evoked activity, while the TTV method continued to indicate a negative correlation. This suggests that our original hypothesis (that the TTV method may indicate spurious negative correlations when there are oscillatory desynchronizations) is still partially correct. Overall, these results underscore the complexity of treating time-domain data in terms of frequency bands. Future work investigating spontaneous-evoked interaction in M/EEG may wish to avoid time-domain analyses entirely, focussing entirely on frequency domain analyses and operationalizing them in terms of both power (as we did in the main text) and phase.

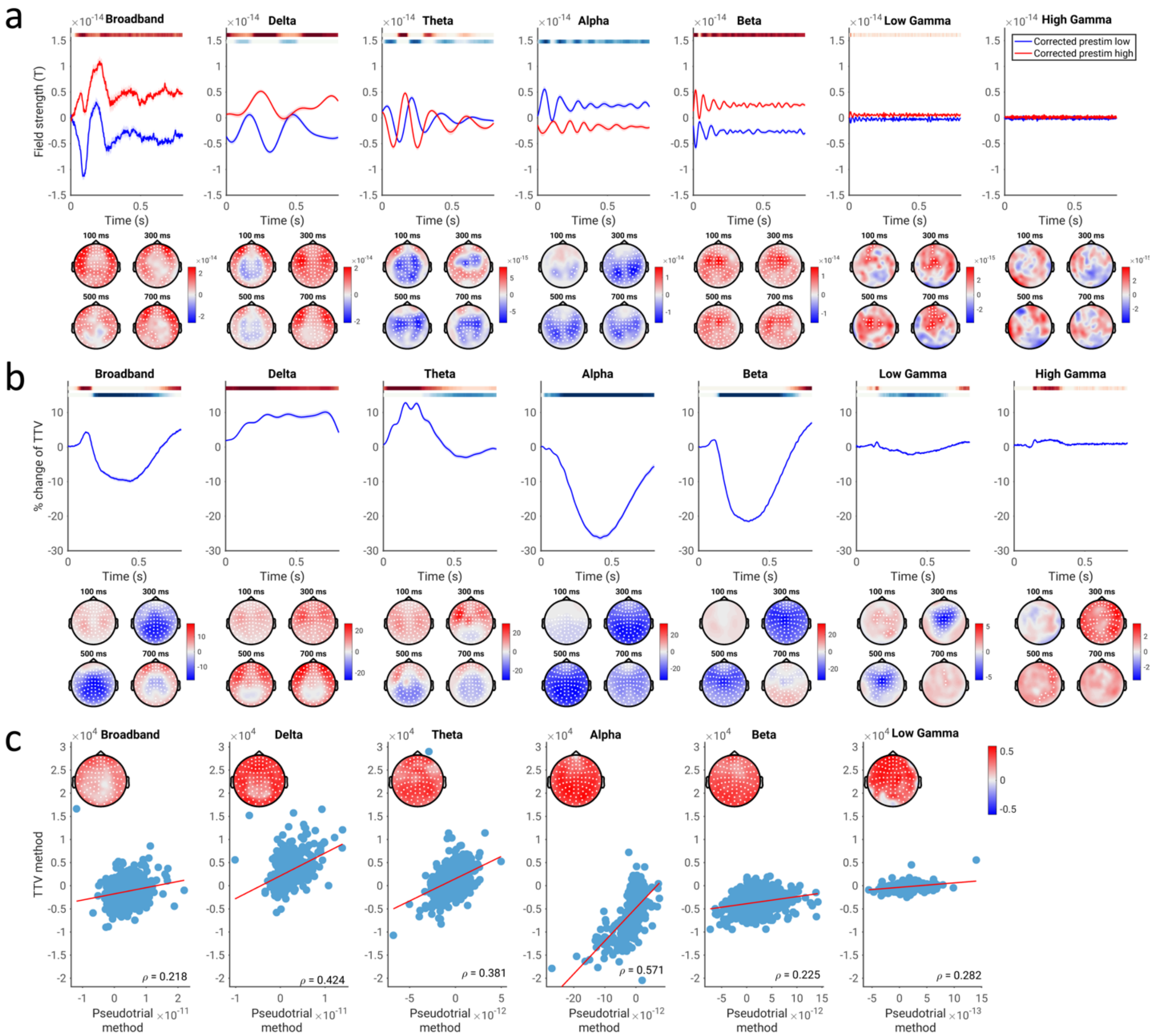

Figure S14: As figure 2 in the main text, but examining time-domain changes in each band, rather than spectral power.

Evaluating potential mechanisms for spontaneous-evoked correlation in the alpha band – the effect of phase-amplitude coupling

Phase-amplitude coupling is a robust phenomenon observed in electrophysiological data in many settings, and between multiple pairs of frequency bands[11]. Phase-amplitude coupling has been associated with learning[12], working memory[13], and perception[14], and has been proposed as a mechanism for connecting large-scale network activity with local computation[11]. Additionally, phase synchronization of low-frequency activity is a phenomenon which has is frequently observed in the context of rhythmic stimulation[15,16], but also as a process of "phase resetting" following non-rhythmic stimuli[17,18].

In fMRI, a previous study showed that the magnitude of phase-amplitude coupling during resting-state predicted the degree of spontaneous-evoked correlation[19]. The combined effects of phase-coherence and phase-amplitude coupling are a potential mechanism for spontaneous-

evoked correlation. If delta phase is coupled to alpha power, then post-stimulus delta phase synchronization could result in a change in alpha power. If phase-synchronization effects were driving this change in alpha power, then this change would be inherently non-additive. Phase synchronization implies some process of phase-resetting, which would result in different responses for different points in the prestimulus delta phase cycle. For example, assume that delta peaks synchronize in the poststimulus period, and the stimulus induces a "resetting" or "jumping" to the peak portion of the phase cycle. For trials where the prestimulus period occurred during the peak of a delta cycle, no change in power would be observed, as the same phase continues to the poststimulus period; on the other hand, for trials where the prestimulus period occurred during the trough of the delta cycle, a maximal change in power would be observed. In this way, the combination of inter-trial phase coherence and phase-amplitude coupling could produce a negative correlation between spontaneous and evoked activity. Though not exhaustively or rigorously, we examined this possibility by assessing delta phase synchronization and its relation to the negative correlation observed in the alpha band.

Inter-trial coherence (ITC) was calculated as described in[20]. Phase-amplitude coupling (PAC) between the delta and alpha bands was assessed using the ERPAC[21]. We then correlated the magnitude of phase-amplitude coupling, the magnitude of inter-trial coherence, and their product with the spontaneous-evoked correlation observed in our data (assessed with the method of pseudotrials). We correlated the PAC and ITC measures with the pseudotrial-corrected difference between prestimulus high and low at each time point, correcting for multiple comparisons with a cluster-based permutation test across time and channels. We also assessed the relationship of PAC and ITC with spontaneous-evoked correlation in a static way: we created a summary index of PAC, ITC, and PAC*ITC by taking the area under the curve across all time points and sensors included in a significant cluster.

We observed significant phase-amplitude coupling at the group level ($p \leq 0.002$), as well as significant inter-trial coherence at the group level ($p \leq 0.002$). The former peaked at 300 ms and remained high, while the latter peaked at 250 ms and returned to baseline. No significant correlations were observed with PAC ($p = 0.5375$), ITC ($p = 0.1139$), or the interaction of the two ($p = 0.5315$) when considering the time-resolved correlations. Considering the summary index correlations, no significant correlations were observed for PAC ($p = 0.1499$) or ITC ($p = 0.3636$); a significant correlation was observed for PAC*ITC, but it was in the opposite direction as expected ($p = 0.0460$).

Though we did not investigate phase-amplitude coupling systematically as a mechanism for spontaneous-evoked correlation, these preliminary results indicate that at least for spontaneous-evoked correlation in alpha, delta-alpha phase-amplitude coupling is not a mechanism. Future research should investigate this hypothesis more thoroughly, as well as other potential mechanisms for spontaneous-evoked correlation.

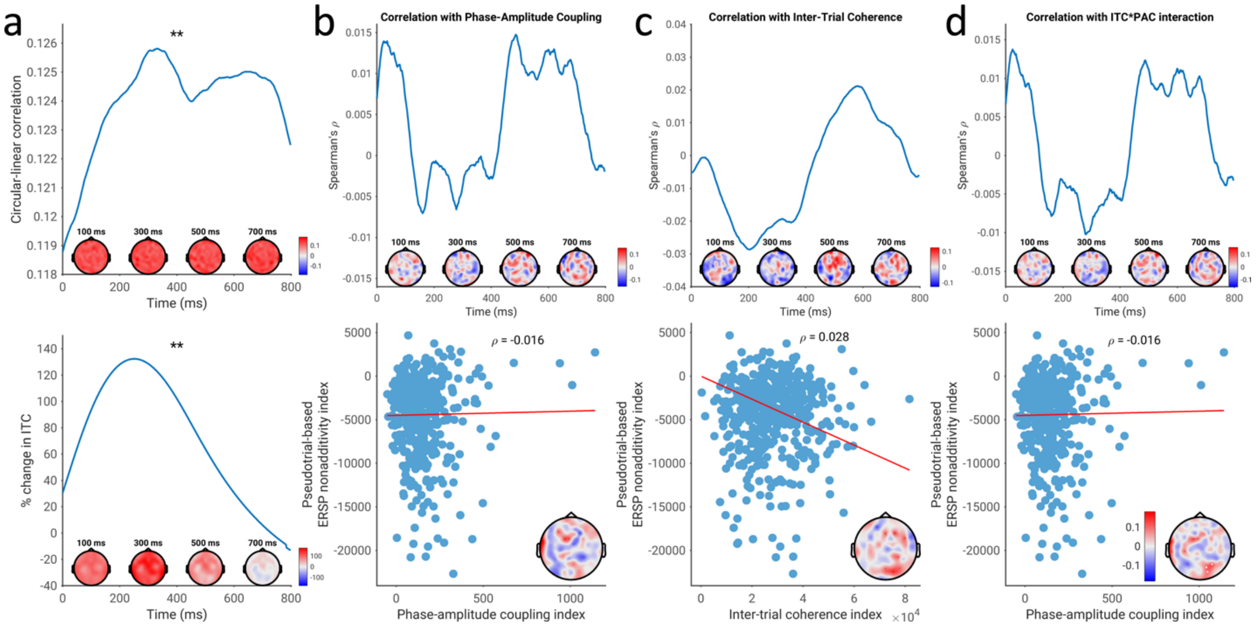

Figure S15: Effect of delta phase coherence and delta-alpha phase amplitude coupling on spontaneous-evoked correlation in the alpha band. **a**) (top) Phase-amplitude coupling between delta and alpha bands, averaged across subjects. Topoplots show the topography of the effect at the indicated latencies. Asterisks indicate significant difference from zero. * = $p < 0.05$, ** = $p < 0.01$. (bottom) Inter-trial coherence in the delta band, expressed as percent change from baseline. **b**) (top) Correlation at each time point between delta-alpha phase amplitude coupling and the magnitude of spontaneous-evoked correlation in alpha, assessed with the method of pseudotrials. Topoplots and asterisks as in **a**). (bottom) Correlation of summary indices of phase-amplitude coupling and spontaneous-evoked correlation. Scatter plots shows the correlation of the mean index across all sensors, while the topoplots show the topographical distribution of the correlation. White dots indicate sensors which are significant following a cluster-based permutation test. **c**) As **b**), but for inter-trial coherence instead of phase-amplitude coupling. **d**) As **b**), but for the interaction of PAC and ITC.

Behavioural implications of the correlation between spontaneous and evoked activity

While the main focus of our paper was on the relationship between spontaneous brain activity and task-evoked brain activity, we also considered the relationship between spontaneous activity and behaviour. As discussed in the introduction, this has been reported more extensively than the relationship between spontaneous and evoked activity[22–26]. We leveraged our datasets to examine two behavioural implications of our data. First, we set out to confirm previously reported relationships between prestimulus power and reaction times[27–32] in a larger dataset. Second, we analyzed whether patterns of spontaneous-evoked correlation differ between different conditions in the moral reasoning task of our EEG dataset.

To address our first question, we fit linear mixed models to predict reaction times in the CamCAN dataset from prestimulus power. We constructed models at each electrode for each frequency band in which we observed significant spontaneous-evoked correlation in our data (i.e. all bands but high gamma). Models included a fixed effect for prestimulus power, as well as a random slope and intercept for each subject. Because performing a permutation test with mixed-effects models was computationally expensive, we instead corrected the p values of the fixed-

effects coefficients in each band for multiple comparisons using FDR correction[33]. We observed significant prediction of reaction time from prestimulus power in all bands. Positive correlations were observed between prestimulus power in delta and theta and reaction time, while negative correlations were observed in alpha, beta, and low gamma. We also fit the same model including poststimulus change in each band (calculated as the area under the curve of the percent change of spectral power): the opposite pattern was observed for these poststimulus change indices. Notably, despite the large alpha response observed in our data, prediction of reaction time was weakest in the alpha band for both the prestimulus and poststimulus models: this is relevant in the context of discrepant findings in small-sample studies in the literature, which have sometimes reported roles for prestimulus alpha in predicting response times[30], but other times have found no such evidence[34]. Overall, these findings confirm widespread reports of prestimulus power in various frequencies being predictive of behavioural reaction times.

To address our second question, we estimated spontaneous-evoked correlation using the methods in the main text separately for the moral judgement and control (counting) conditions in the task from[35], focusing on the frequency-domain data. We then compared the estimates of spontaneous-evoked correlation by comparing the pseudotrial-corrected prestim high minus low time courses in each condition, and by comparing the TTV time courses, using Wilcoxon signed rank tests with cluster correction as done in the main text to compare spontaneous-evoked correlation of oscillatory and fractal dynamics. We observed no differences between the conditions (with the exception of a small difference in gamma, where the counting condition displayed a larger negative correlation between spontaneous and evoked gamma: $p = 0.006$, average $d = 0.514$). This result further supports the assumption that correlation between spontaneous and evoked activity in spectral power reflects a task-independent dynamic process.

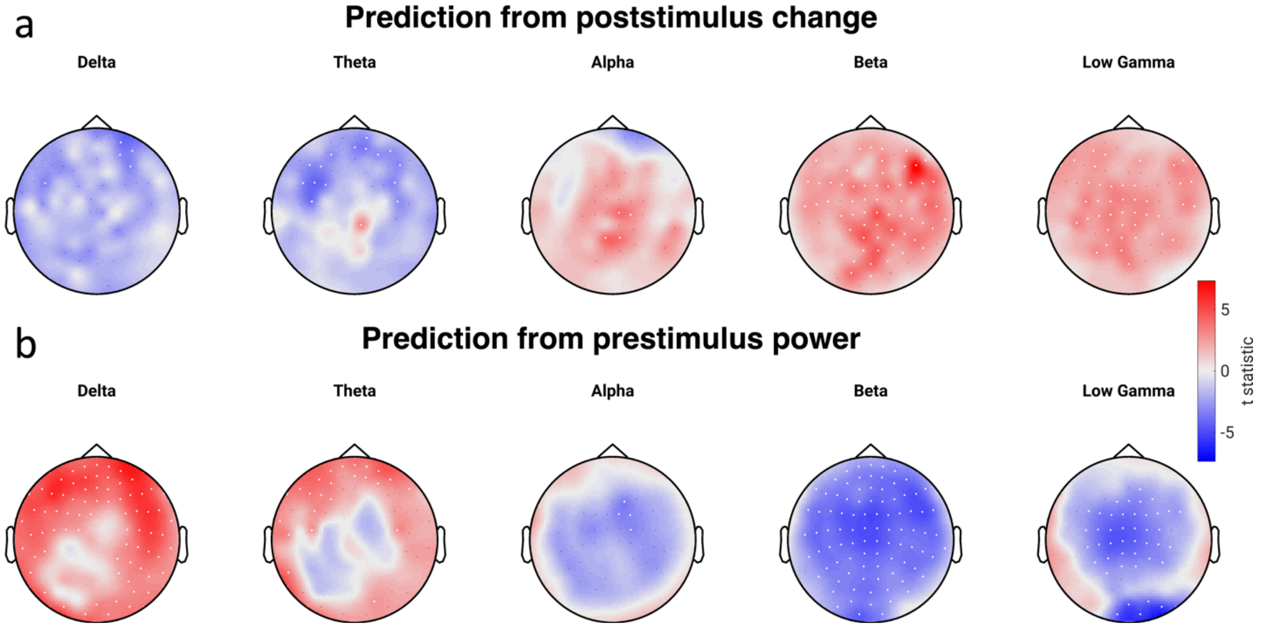

Figure S16: Prediction of reaction time in the CamCAN dataset from **a**) poststimulus change, calculated as the area under the curve of the percent change of power in each band, and **b**) prestimulus power. Topographies show the t-statistic for the fixed effect each parameter: white electrodes indicate sensors which were significant at FDR < 0.05.

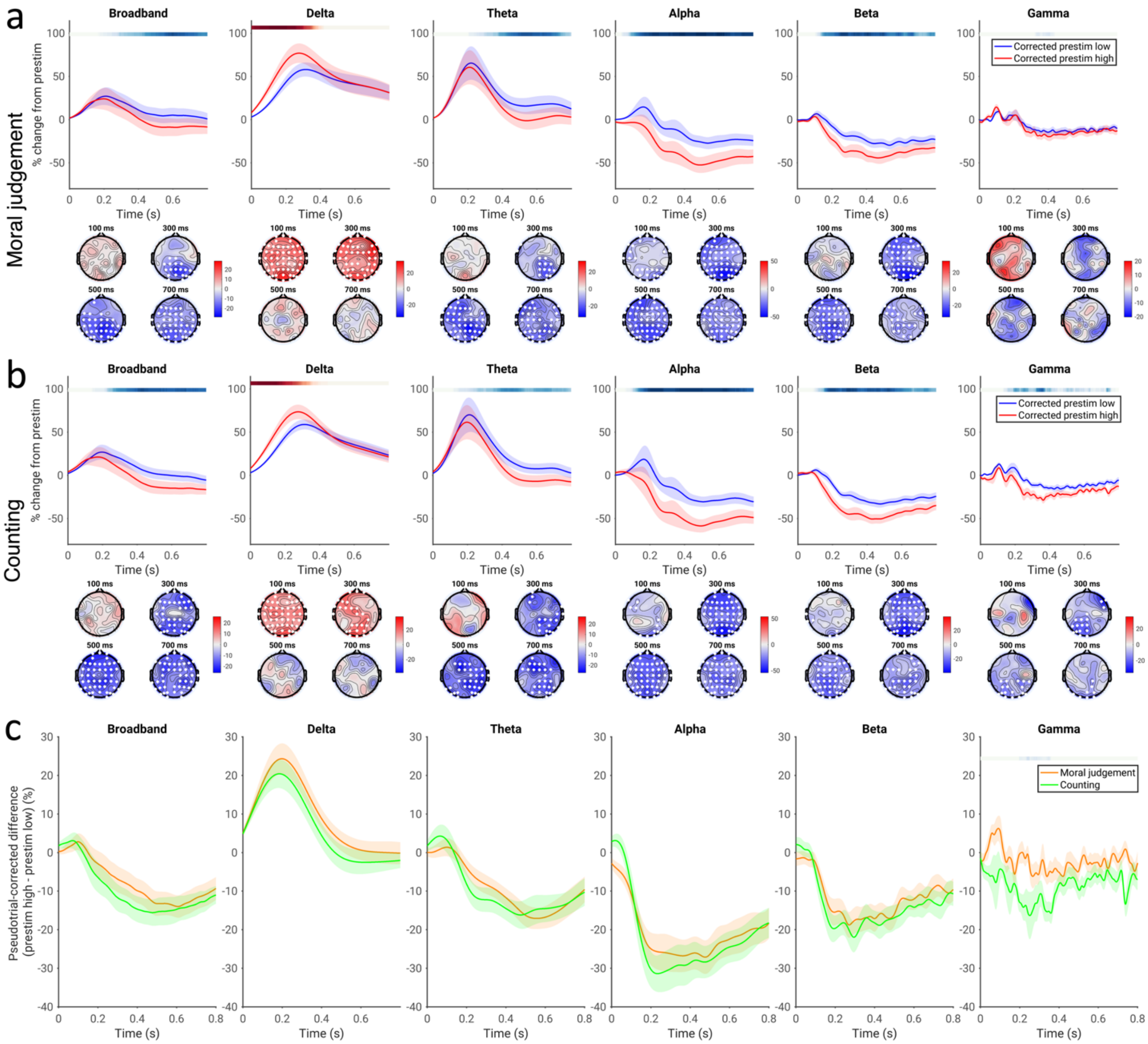

Figure S17: Comparison of spectral power spontaneous-evoked correlation between the moral judgement and counting conditions of the task in Wolff et al. Conventions as figure S11.

Supplementary references

1. Muthukumaraswamy, S. D. & Liley, D. TJ. 1/f electrophysiological spectra in resting and drug-induced states can be explained by the dynamics of multiple oscillatory relaxation processes. *NeuroImage* **179**, 582–595 (2018).
2. Ouyang, G., Hildebrandt, A., Schmitz, F. & Herrmann, C. S. Decomposing alpha and 1/f brain activities reveals their differential associations with cognitive processing speed. *NeuroImage* **205**, 116304 (2020).
3. Kutas, M. & Federmeier, K. D. Thirty years and counting: finding meaning in the N400 component of the event-related brain potential (ERP). *Annu Rev Psychol* **62**, 621–647 (2011).
4. Goregliad Fjaellingsdal, T. *et al.* Expectancy effects in the EEG during joint and spontaneous word-by-word sentence production in German. *Scientific Reports* **10**, 5460 (2020).
5. Racz, F. S. *et al.* Separating scale-free and oscillatory components of neural activity in schizophrenia. *Brain and Behavior* **n/a**, e02047 (2021).
6. Tanner, D., Morgan-Short, K. & Luck, S. J. How inappropriate high-pass filters can produce artifactual effects and incorrect conclusions in ERP studies of language and cognition. *Psychophysiology* **52**, 997–1009 (2015).
7. Winkler, I., Debener, S., Müller, K.-R. & Tangermann, M. On the influence of high-pass filtering on ICA-based artifact reduction in EEG-ERP. *Conf Proc IEEE Eng Med Biol Soc* **2015**, 4101–4105 (2015).
8. Arazi, A., Censor, N. & Dinstein, I. Neural Variability Quenching Predicts Individual Perceptual Abilities. *J. Neurosci.* **37**, 97–109 (2017).

9. Arazi, A., Gonen-Yaacovi, G. & Dinstein, I. The Magnitude of Trial-By-Trial Neural Variability Is Reproducible over Time and across Tasks in Humans. *eNeuro* **4**, ENEURO.0292-17.2017 (2017).

10. Başar-Eroglu, C., Başar, E., Demiralp, T. & Schürmann, M. P300-response: Possible psychophysiological correlates in delta and theta frequency channels: A review. *International Journal of Psychophysiology* **13**, 161–179 (1992).

11. Canolty, R. T. & Knight, R. T. The functional role of cross-frequency coupling. *Trends Cogn. Sci. (Regul. Ed.)* **14**, 506–515 (2010).

12. Tort, A. B. L., Komorowski, R. W., Manns, J. R., Kopell, N. J. & Eichenbaum, H. Theta–gamma coupling increases during the learning of item–context associations. *PNAS* **106**, 20942–20947 (2009).

13. Axmacher, N. *et al.* Cross-frequency coupling supports multi-item working memory in the human hippocampus. *PNAS* **107**, 3228–3233 (2010).

14. Fiebelkorn, I. C. *et al.* Cortical cross-frequency coupling predicts perceptual outcomes. *NeuroImage* **69**, 126–137 (2013).

15. Lakatos, P., Schroeder, C. E., Leitman, D. I. & Javitt, D. C. Predictive Suppression of Cortical Excitability and Its Deficit in Schizophrenia. *J Neurosci* **33**, 11692–11702 (2013).

16. Luo, H. & Poeppel, D. Phase patterns of neuronal responses reliably discriminate speech in human auditory cortex. *Neuron* **54**, 1001–1010 (2007).

17. Makeig, S. *et al.* Dynamic Brain Sources of Visual Evoked Responses. *Science* **295**, 690–694 (2002).

18. Barry, R. J. Evoked activity and EEG phase resetting in the genesis of auditory Go/NoGo ERPs. *Biological Psychology* **80**, 292–299 (2009).

19. Huang, Z. *et al.* Is There a Nonadditive Interaction Between Spontaneous and Evoked Activity? Phase-Dependence and Its Relation to the Temporal Structure of Scale-Free Brain Activity. *Cereb. Cortex* **27**, 1037–1059 (2017).

20. Delorme, A. & Makeig, S. EEGLAB: an open source toolbox for analysis of single-trial EEG dynamics including independent component analysis. *J. Neurosci. Methods* **134**, 9–21 (2004).

21. Voytek, B., D'Esposito, M., Crone, N. & Knight, R. T. A method for event-related phase/amplitude coupling. *Neuroimage* **64**, 416–424 (2013).

22. Benwell, C. S. Y. *et al.* Prestimulus EEG Power Predicts Conscious Awareness But Not Objective Visual Performance. *eNeuro* **4**, (2017).

23. Sadaghiani, S., Hesselmann, G., Friston, K. J. & Kleinschmidt, A. The Relation of Ongoing Brain Activity, Evoked Neural Responses, and Cognition. *Front Syst Neurosci* **4**, (2010).

24. Sadaghiani, S., Poline, J.-B., Kleinschmidtc, A. & D'Esposito, M. Ongoing dynamics in large-scale functional connectivity predict perception. *Proceedings of the National Academy of Sciences of the United States of America* **112**, 8463–8468 (2015).

25. Supèr, H., Togt, C. van der, Spekreijse, H. & Lamme, V. A. F. Internal State of Monkey Primary Visual Cortex (V1) Predicts Figure–Ground Perception. *J. Neurosci.* **23**, 3407–3414 (2003).

26. Bai, Y. *et al.* Resting state glutamate predicts elevated pre-stimulus alpha during self-relatedness: A combined EEG-MRS study on "rest-self overlap". *Social Neuroscience* **11**, 249–263 (2016).

27. Reinhart, R. M. G., Mathalon, D. H., Roach, B. J. & Ford, J. M. Relationships between pre-stimulus gamma power and subsequent P300 and reaction time breakdown in schizophrenia. *International Journal of Psychophysiology* **79**, 16–24 (2011).

28. Bompas, A., Sumner, P., Muthumumaraswamy, S. D., Singh, K. D. & Gilchrist, I. D. The contribution of pre-stimulus neural oscillatory activity to spontaneous response time variability. *NeuroImage* **107**, 34–45 (2015).

29. Besserve, M. *et al.* Prediction of performance level during a cognitive task from ongoing EEG oscillatory activities. *Clinical Neurophysiology* **119**, 897–908 (2008).

30. van den Berg, B., Appelbaum, L. G., Clark, K., Lorist, M. M. & Woldorff, M. G. Visual search performance is predicted by both prestimulus and poststimulus electrical brain activity. *Scientific Reports* **6**, 37718 (2016).

31. Chen, Y. *et al.* The Weakened Relationship Between Prestimulus Alpha Oscillations and Response Time in Older Adults With Mild Cognitive Impairment. *Front. Hum. Neurosci.* **14**, (2020).

32. Klein, C. *et al.* The Influence of Pre-stimulus EEG Activity on Reaction Time During a Verbal Sternberg Task is Related to Musical Expertise. *Brain Topogr* **29**, 67–81 (2016).

33. Benjamini, Y. & Yekutieli, D. The control of the false discovery rate in multiple testing under dependency. *Ann. Statist.* **29**, 1165–1188 (2001).

34. van Dijk, H., Schoffelen, J.-M., Oostenveld, R. & Jensen, O. Prestimulus oscillatory activity in the alpha band predicts visual discrimination ability. *J. Neurosci.* **28**, 1816–1823 (2008).

35. Wolff, A. *et al.* Neural variability quenching during decision-making: Neural individuality and its prestimulus complexity. *Neuroimage* **192**, 1–14 (2019).
