# Supplementary material for: Dynamic relationships between spontaneous and evoked electrophysiological activity
Source: Commun Biol. 2021 Jun 15;4:741. doi: 10.1038/s42003-021-02240-9 (PMC8206204; doi:10.1038/s42003-021-02240-9)
Supplement: Supplementary file 2 — Reporting Summary [file 42003_2021_2240_MOESM2_ESM.pdf]

nature research

Corresponding author(s): Georg Northoff
Last updated by author(s): Apr 29, 2021

Reporting Summary

Nature Research wishes to improve the reproducibility of the work that we publish. This form provides structure for consistency and transparency in reporting. For further information on Nature Research policies, see our Editorial Policies and the Editorial Policy Checklist.

Statistics

For all statistical analyses, confirm that the following items are present in the figure legend, table legend, main text, or Methods section.

n/a	Confirmed	
☐	☒	The exact sample size (n) for each experimental group/condition, given as a discrete number and unit of measurement
☐	☒	A statement on whether measurements were taken from distinct samples or whether the same sample was measured repeatedly
☐	☒	The statistical test(s) used AND whether they are one- or two-sided<br>*Only common tests should be described solely by name; describe more complex techniques in the Methods section.*
☐	☒	A description of all covariates tested
☐	☒	A description of any assumptions or corrections, such as tests of normality and adjustment for multiple comparisons
☐	☒	A full description of the statistical parameters including central tendency (e.g. means) or other basic estimates (e.g. regression coefficient) AND variation (e.g. standard deviation) or associated estimates of uncertainty (e.g. confidence intervals)
☐	☒	For null hypothesis testing, the test statistic (e.g. F, t, r) with confidence intervals, effect sizes, degrees of freedom and P value noted<br>*Give P values as exact values whenever suitable.*
☒	☐	For Bayesian analysis, information on the choice of priors and Markov chain Monte Carlo settings
☒	☐	For hierarchical and complex designs, identification of the appropriate level for tests and full reporting of outcomes
☐	☒	Estimates of effect sizes (e.g. Cohen's d, Pearson's r), indicating how they were calculated

Our web collection on statistics for biologists contains articles on many of the points above.

Software and code

Policy information about availability of computer code

Data collection	Study is a re-analysis; as such, no data was collected specifically for the study. Information about data collection for the CamCAN MEG dataset is described in Shafto et al. (2014) and Taylor et al. (2017). Information about data collection for the EEG dataset is provided in Wolff et al. (2019).
Data analysis	Data were analyzed in MATLAB R2016b and R2019a. Fieldtrip (downloaded October 2018), EEGLAB (v14.1.2b), and MNE-Python (v0.20.3) were used in data analysis. Custom scripts were used for the analysis of spontaneous-evoked interaction; these are available from GitHub (https://github.com/SorenWT/spontevo2020).

For manuscripts utilizing custom algorithms or software that are central to the research but not yet described in published literature, software must be made available to editors and reviewers. We strongly encourage code deposition in a community repository (e.g. GitHub). See the Nature Research guidelines for submitting code & software for further information.

Data

Policy information about availability of data

All manuscripts must include a data availability statement. This statement should provide the following information, where applicable:
- Accession codes, unique identifiers, or web links for publicly available datasets
- A list of figures that have associated raw data
- A description of any restrictions on data availability

MEG data are available from the CamCAN repository (https://camcan-archive.mrc-cbu.cam.ac.uk/dataaccess/). Data from the EEG replication study are available from the corresponding author on reasonable request.

Field-specific reporting

Please select the one below that is the best fit for your research. If you are not sure, read the appropriate sections before making your selection.

☒ Life sciences ☐ Behavioural & social sciences ☐ Ecological, evolutionary & environmental sciences

For a reference copy of the document with all sections, see nature.com/documents/nr-reporting-summary-flat.pdf

Life sciences study design

All studies must disclose on these points even when the disclosure is negative.

Sample size	Study was a re-analysis of existing datasets; sample size was thus subject to these constraints.
Data exclusions	In a previous version of this project, we also analyzed resting-state recordings and their relation to spontaneous-evoked interaction; these findings will now be reported in a forthcoming publication. As a result, we analyzed only participants whose task recording had a corresponding resting-state recording. This resulted in the exclusion of 74 participants from the CamCAN MEG study, and 8 participants from the Wolff et al. EEG study.
Replication	An independent EEG dataset was used to replicate the original MEG findings; the results of this are described in the main text of the article.
Randomization	Not applicable; participants were not divided into groups in the study.
Blinding	Not applicable; participants were not divided into groups in the study.

Reporting for specific materials, systems and methods

We require information from authors about some types of materials, experimental systems and methods used in many studies. Here, indicate whether each material, system or method listed is relevant to your study. If you are not sure if a list item applies to your research, read the appropriate section before selecting a response.

Materials & experimental systems

n/a	Involved in the study	
☒	☐	Antibodies
☒	☐	Eukaryotic cell lines
☒	☐	Palaeontology and archaeology
☒	☐	Animals and other organisms
☐	☒	Human research participants
☒	☐	Clinical data
☒	☐	Dual use research of concern

Methods

n/a	Involved in the study	
☒	☐	ChIP-seq
☒	☐	Flow cytometry
☒	☐	MRI-based neuroimaging

Human research participants

Policy information about studies involving human research participants

Population characteristics	CamCAN main dataset (n = 474). Age: M = 56.54, SD = 15.56. Sex: 234 females. Handedness: 432 right-handed.<br>CamCAN IRASA subset (n = 49): Age: M = 45.62, SD = 11.89. Sex: 28 females. Handedness: 44 right-handed.<br>EEG replication dataset (n = 26). Age: M = 29.50, SD = 11.06. Sex: 15 females. Handedness: all right-handed.
Recruitment	See Shafto et al. (2014) and Taylor et al. (2017) for recruitment information for the MEG dataset. See Wolff et al. (2019) for recruitment information for the EEG dataset.
Ethics oversight	CamCAN dataset: Cambridgeshire 2 Research Ethics Committee (reference: 10/H0308/50)<br>Wolff et al. EEG dataset: University of Ottawa Institute of Mental Health Reserach REB (REB # 2009018)

Note that full information on the approval of the study protocol must also be provided in the manuscript.
